# Pbx1 and Pbx3 cooperatively regulate intermediate progenitor genesis and corticogenesis in the mouse neocortex

**DOI:** 10.3389/fcell.2026.1809251

**Published:** 2026-07-02

**Authors:** Asisa Muchamedin, Pauline A. Ulmke, Linh Pham, Hoang Duy Nguyen, Marie-Luise Kümmel, Boris Burr, David Bietz, Petra Wahle, Huu Phuc Nguyen, Tran Tuoc

**Affiliations:** 1 Department of Human Genetics, Ruhr University of Bochum, Bochum, Germany; 2 Department of Neuroanatomy, Ruhr University of Bochum, Bochum, Germany; 3 Department of Developmental Biology, Ruhr University of Bochum, Bochum, Germany

**Keywords:** anterior commissure, corpus callosum, cortical development, interhemispheric connectivity, intermediate progenitor cells, layer formation, neurogenesis, Pbx

## Abstract

Intermediate progenitor cells (IPCs) are key amplifying neuronal precursors that generate the majority of glutamatergic projection neurons during neocortical development. Despite their central role in corticogenesis, the transcriptional mechanisms controlling IPC proliferation and lineage progression remain incompletely defined. Here we combined single-nucleus transcriptomics with bulk RNA sequencing of purified IPCs to identify the TALE homeodomain transcription factors Pbx1 and Pbx3 as prominent regulators in cortical progenitor populations. Single-nucleus RNA-seq of Tbr2^+^ IPCs reveals broad expression of Pbx1 across IPC states, with Pbx3 selectively enriched in proliferative IPCs. Conditional dual deletion of *Pbx1/Pbx3* in the dorsal telencephalon using Emx1-Cre resulted in a marked reduction of proliferating IPCs during embryogenesis, while radial glial cell numbers and survival were largely preserved. At postnatal stages, *Pbx1/Pbx3* double conditional mutants displayed microcephaly with reduced cortical size, disrupted laminar organization, increased numbers of deep-layer neurons, and a selective depletion of upper-layer neurons. These defects were accompanied by severe abnormalities in forebrain connectivity, including complete loss of the anterior commissure and partial agenesis of the corpus callosum. Integrated bulk-RNA-seq and CUT&Tag profiling of IPCs identified a core set of direct Pbx1/Pbx3 transcriptional targets implicated in IPC identity and lineage progression, including *Cux2*, *Insm1*, *Lhx2, Myt1l*, and *Trnp1*. Together, our findings establish Pbx1 and Pbx3 as essential transcriptional regulators of IPC proliferation and differentiation, thereby ensuring proper cortical neuron production and forebrain morphogenesis.

## 1 Introduction

The development of the mammalian cerebral cortex depends on tightly coordinated processes of neural stem and progenitor cell proliferation, migration, and differentiation, that together generate the diverse neuronal populations underlying higher cognitive functions. Among eutherian mammals, the neocortex and the corpus callosum represent the most evolutionarily recent brain structures (Mihrshahi, 2006; Gavrish et al., 2023). Evolutionary variations in cortical morphology, from the smooth lissencephalic rodent cortex to the highly folded gyrencephalic human cortex, reflect differences in neurogenic programs that drive cortical expansion. A key component of these programs is the population of intermediate progenitor cells (IPCs), transit amplifying cells expressing the T-box transcription factor Tbr2/Eomes. IPCs emerge through asymmetric divisions of radial glial cells (RGCs) in the ventricular zone (Haubensak et al., 2004; Kriegstein and Alvarez-Buylla, 2009). IPCs migrate to the subventricular zone, where they generate the majority of glutamatergic projection neurons in both rodents and primates (Götz and Huttner, 2005; Martínez-Cerdeño, et al., 2006).

**FIGURE 1:**
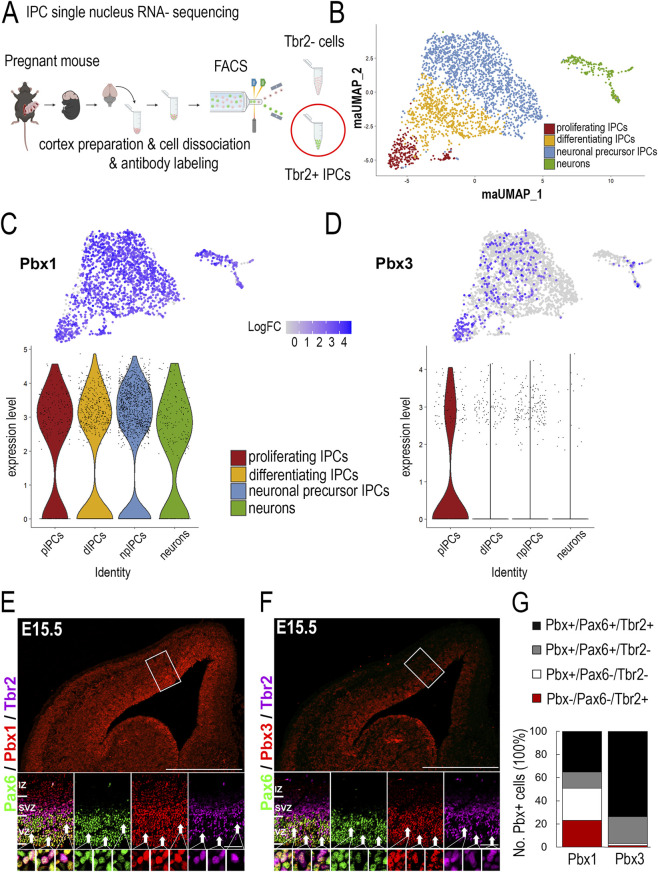
Pbx1 and Pbx3 are expressed in intermediate progenitor cells (IPCs) during forebrain development. **(A)** Schematic of the workflow for nuclei isolation and single-nucleus RNA sequencing (created with BioRender). **(B)** UMAP plots of the four major cell populations: proliferating IPCs, differentiating IPCs, neuronal precursor IPCs, and neurons. **(C,D)** Violin plots showing Pbx1 and Pbx3 expression across cell types. **(E,F)** Triple immunohistochemistry of control cortices at E15.5 using antibodies against Pax6, Tbr2 and Pbx1 or Pbx3. Representative middle sections are shown. White arrows indicate triple-positive cells. Cell counts were obtained using CellProfiler. Scale bars: 500 µm (overview) and 50 µm (higher magnification). **(G)** Proportion of Pbx1^+^ and Pbx3^+^ cells co-expressing Pax6, Tbr2, or both Pax6/Tbr2**.** FACS, fluorescence-activated cell sorting.

Our previous studies (Narayanan et al., 2018; Kerimoglu et al., 2021; Ulmke et al., 2021) revealed substantial heterogeneity within the IPC population, with distinct subsets differing in lineage potential and responsiveness to extracellular cues. This heterogeneity contributes to cortical neuronal diversity and underscores that IPCs are not merely a transient population but actively shape cortical architecture. Single-nucleus transcriptomics has further resolved the molecular diversity within IPCs, uncovering previously unrecognized subpopulations and transcriptional programs that govern their developmental trajectories and neurogenic capacity (Ulmke et al., 2025). Among the transcriptional regulators shaping IPC identity, members of the PBX family of TALE homeodomain transcription factors, particularly pre-B-cell leukemia transcription factor 1 (Pbx1) and Pbx3, have emerged as factors of interest (Figures 1A–D). Pbx proteins belong to the superclass of the Three-Amino-acid-Loop-Extension-homeodomain (TALE-HD) transcription factor family. They are evolutionarily conserved and contain an atypical homeodomain (Liu et al., 2022; Crisafulli et al., 2024). As components of multiple gene regulatory networks, Pbx transcription factors orchestrate diverse aspects of embryogenesis, including patterning of the limbs and the main anterior-posterior body-axis (Selleriet al., 2019), by modulating gene expression programs that govern cell proliferation, differentiation and apoptosis (Grebbin and Schulte, 2017). Beyond their developmental roles, Pbx transcription factors are essential for the morphogenesis of multiple organs and contribute to hematopoiesis and stem cell pluripotency (Zewdu et al., 2016; Liu et al., 2022). In the developing forebrain, Pbx1 promotes rostral cortical identity in early progenitors, thereby influencing the establishment of the rostrocaudal and dorsoventral patterning within the neocortex. This function positions Pbx1 as a key transcriptional regulator of cortical arealization rather than a general neurogenic determinant (Golonzhka et al., 2015). In the adult brain, Pbx1 remains critical for neural stem and progenitor cell fate, promoting neurogenic differentiation and guiding appropriate tangential migration (Grebbin et al., 2016; Hau et al., 2021; Laub et al., 2024). Pbx3 primarily acts as a transcriptional cofactor, frequently forming complexes with Hox/Meis proteins, and plays a pivotal role in hindbrain development and regulation of respiratory neural circuits (Rhee et al., 2004; Rottkamp et al., 2008).

Clinically, Pbx1 haploinsufficiency has been linked to a variety of neurodevelopmental disorders, including microcephaly and autism-spectrum disorders (Slavotinek et al., 2017; Fitzgerald et al., 2021; Crisafulli et al., 2024), whereas Pbx3 has been clinically linked to the development of glioma (Zhao et al., 2024). Disruption of Pbx-dependent transcriptional programs is likely to impair neuronal subtype specification and long-range connectivity and may contribute to an altered excitatory-inhibitory balance in the developing cortex (Golonzhka et al., 2015; Hanley et al., 2016). These insights underscore the critical role of *Pbx1/Pbx3* in cortical and postnatal brain maturation and suggest that Pbx1/Pbx3 related pathologies may represent a mechanistic link between impaired progenitor dynamics and higher order-cognitive dysfunction. Together, these findings motivate a focused investigation into how Pbx1/Pbx3 transcription factors regulate IPC identity, lineage progression, and the establishment of functional cortical circuits.

Here, we perform a loss-of-function study in transgenic mice carrying conditional *Pbx1* and *Pbx3* alleles, which were selectively deleted in the telencephalon using *Emx1-*Cre (Gorski et al., 2002). Double conditional knockout (dcKO) animals exhibited a reduced brain size, laminar disorganization, impaired interhemispheric connectivity, loss of the anterior commissure and a partial agenesis of the corpus callosum. Transcriptomic profiling combined with integrated CUT&Tag analysis identified novel Pbx1/Pbx3 target candidate genes, including *Cux2, Insm1, Lhx2, Myt1l*, and *Trnp1*.

A fundamental question in developmental neurobiology is how transcription factors generate heterogeneity among neural progenitor populations and control cell fate decisions. Despite evidence that *Pbx1/Pbx3* are critical for cortical patterning and neurogenesis, it remains unclear how they influence IPC cell fate decisions, cortical lamination, and interhemispheric connectivity. Here, we use *Emx1*-Cre–mediated conditional inactivation of *Pbx1/Pbx3* in dorsal telencephalic progenitors, combined with transcriptomic and chromatin profiling, to define *Pbx1/Pbx3*-dependent gene regulatory networks in IPCs. We hypothesize that *Pbx1/Pbx3* cooperatively control IPC abundance and identity, thereby affecting upper- and deep-layer neuron production and the formation of callosal and commissural structures through regulation of IPC-associated transcriptional programs.

## 2 Results

### 2.1 Single-nucleus transcriptomics identifies Pbx transcription factors in intermediate progenitor cell regulation

To investigate transcriptional heterogeneity and regulatory programs in cortical intermediate progenitor cells (IPCs), we applied single-nucleus RNA sequencing (snRNA-seq) to Tbr2^+^ cells isolated from embryonic mouse cortex. We used our previously established intranuclear immunofluorescence protocol targeting Tbr2, followed by fluorescence-activated cell sorting (FACS) (Sakib et al., 2021; Ulmke et al., 2021), to enrich IPC nuclei from embryonic day E16.5 cortices (Figure 1A) (Ulmke et al., 2025). Unsupervised clustering revealed four transcriptionally distinct populations: proliferating IPCs (pIPCs), differentiating IPCs (dIPCs), neuronal precursor IPCs (npIPCs) and neurons, each characterized by a specific gene expression signature, reflecting progressive stages along the IPC to neuron differentiation trajectory (Figure 1B). Transcriptomic profiling revealed a high expression of *Pbx1* across all four clusters, while *Pbx3* expression was significantly enriched in the pIPC cluster (p < 0.001, log_2_FC = 1.67) (Figures 1C,D).

To validate these transcriptomic findings, we performed triple immunohistochemical (IHC) staining on coronal forebrain sections from E15.5 embryos using antibodies against Pbx1 or Pbx3, together with Tbr2 (IPC marker) and Pax6 (radial glia cell marker) (Figures 1E,F). Pbx1 was broadly expressed in cortical progenitor cells within the ventricular and subventricular zone (VZ/SVZ) with markedly lower expression in the intermediate zone (IZ) and cortical plate (CP) (Figure 1E). In contrast, Pbx3 expression was largely restricted to progenitors in the VZ/SVZ (Figure 1F). Quantitative analysis of immunostained sections showed that the majority of Pbx1^+^ or Pbx3^+^ cells co-expressed Tbr2 and Pax6 (Figure 1G), confirming their association with IPCs and RGCs (Figures 1E,F, high magnification images with white arrows indicate triple labeled cells).

Together, these results identify Pbx1 and Pbx3 as prominent transcription factors expressed in cortical progenitor populations and suggest a particular association of Pbx3 with proliferative IPC states.

### 2.2 Conditional deletion of Pbx1 and Pbx3 reduces pool of intermediate progenitor cells

To investigate the roles of *Pbx1*/*Pbx3* in cortical development, with a particular focus on IPC generation, we crossed mice carrying floxed alleles of *Pbx1* and *Pbx3* with cortex-specific *Emx1*-Cre-mice (Gorski et al., 2002). This strategy generated single conditional knockout (*Pbx1*-scKO and *Pbx3*-scKO) and double conditional knockout (*Pbx1/Pbx3*-dcKO) mutant lines. At E15.5, immunohistochemical analysis confirmed that expression of *Pbx1* and *Pbx3* remained detectable in *Pbx1-*and *Pbx3-*scKO cortices (Supplementary Figures S1A,B). In contrast, both *Pbx1* and *Pbx3* expressions were efficiently abolished in dcKO cortices (Supplementary Figure S1C).

**FIGURE 2:**
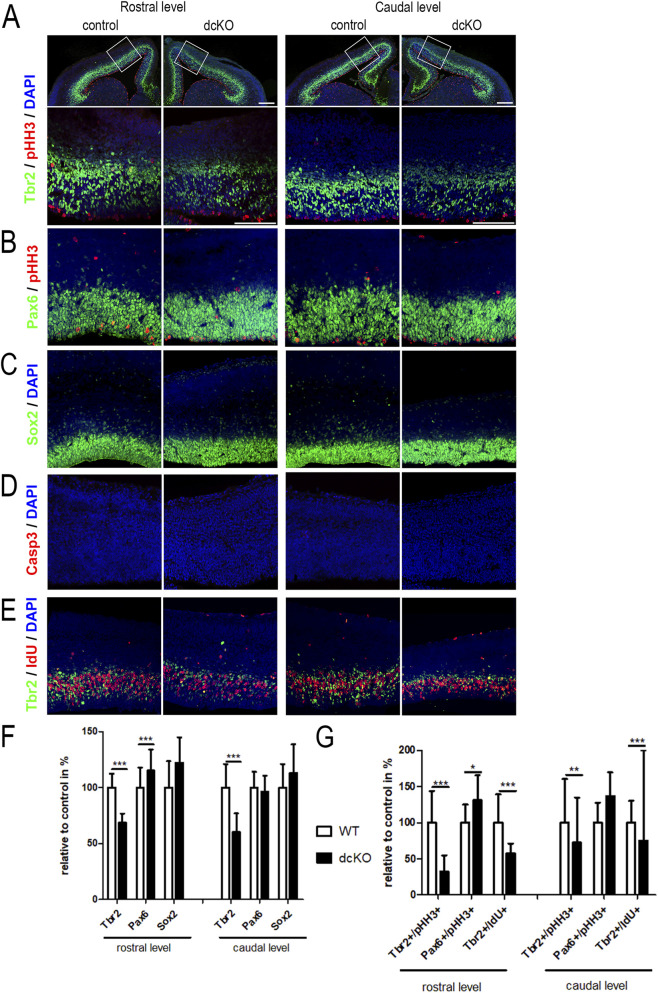
Pbx1/Pbx3 double knockout reduces the pool of intermediate progenitor cells (IPCs) in the neocortex. **(A–E)** Double immunohistochemical staining of rostral and caudal coronal forebrain sections from Pbx1/Pbx3 dcKO and control littermates at E15.5, shown at low (scale bar, 100 µm) and higher magnification (scale bar, 50 µm), as indicated by white frames. **(F,G)** Quantification of Tbr2^+^, Pax6^+^, Sox2^+^, Tbr2^+^/pHH3^+^, Pax6^+^/pHH3^+^ and IdU^+^/Tbr2^+^ cells in dcKO and control cortices. Quantification revealed a reduced number of Tbr2+ IPCs **(A)**, accompanied by increased numbers of Pax6^+^ cells at rostral levels **(B)** and elevated numbers of Sox2^+^ **(C)** radial glial cells. No difference in apoptosis was observed between dcKO and control embryos, as assessed by Casp3 staining **(D)**. A reduced proportion of Tbr2^+^/pHH3^+^ but an increased proportion of Pax6^+^/pHH3^+^ cells was detected in dcKO animals. **(F)** Data represent mean ± SD from three biological replicates, at least four images per replicate were used for quantification (n = 3, 12). **(G)** Data represent mean ± SD from five biological replicates, at least four images per replicate were used for quantification (n = 5, 20). Cell counts were obtained using CellProfiler. Statistical analysis was performed with Mann-Whitney-U test: p < 0.05, **p < 0.01, ***p < 0.001.

To assess the impact of *Pbx1/Pbx3* loss on progenitor populations, coronal forebrain sections from dcKO and control embryos were analyzed, at both rostral and caudal levels using markers for IPCs and radial glial cells (RGCs). Immunostaining for Tbr2 revealed a pronounced reduction in Tbr2^+^ IPCs within the subventricular zone (SVZ) of dcKO cortices at both rostro-caudal levels compared with controls (Figures 2A,F). Quantitative IHC analysis on Tbr2^+^ IPCs in *Pbx1*-scKO and *Pbx3*-scKO determined no significant changes in Tbr2 population compared to controls (Supplementary Figures SD–F). As single conditional mutants did not show obvious changes in the number of Tbr2^+^ IPCs, subsequent analyses focused on dcKO mutants. The number of Pax6^+^ and Sox2^+^ RGCs in the ventricular zone (VZ) was largely unchanged, with the exception of an increase of Pax6^+^ cells in the rostral cortex of dcKO embryos (Figures 2B,C,F).

To determine whether increased cell death contributed to the reduction of IPCs, we examined cleaved Caspase-3 expression. No overt differences in apoptotic cell numbers were observed between dcKO and control cortices (Figure 2D), indicating that enhanced cell death is unlikely to be the primary cause of IPC depletion. However, the current data do not distinguish between impaired IPC generation, premature cell cycle exit, or altered IPC identity, and multiple mechanisms may contribute to the observed phenotype.

We next examined proliferation and mitotic activity. Pregnant females were administered the thymidine analogs CIdU (at E14.5 for 24 h) and IdU (23 h later, for 1 h) prior to tissue collection (Supplementary Figure S2A). Sections were immunostained for IdU (marker of S-phase cells) and phospho-histone H3 (pHH3, marker for mitotic cells) (Figure 2E). We did not observe any difference in the total number of pHH3^+^ cells between dcKO and control cortices (Supplementary Figure S2B), suggesting that total mitotic activity was preserved. However, cell-type-specific analyses revealed marked differences.

Quantification of Tbr2^+^/pHH3^+^ double-positive cells revealed a significant reduction in proliferating IPCs in dcKO cortices, with a more pronounced effect at rostral levels, consistent with the spatial activity of *Emx1-*Cre (Figures 2A,G). Furthermore, the fraction of Tbr2^+^/IdU^+^ double-positive cells was significantly reduced in dcKO cortices compared to controls (Figures 2E,G). In contrast, the number of Pax6^+^/pHH3^+^ double-positive RGCs was modestly increased in dcKO cortices (Figures 2B,G), further supporting impaired IPC production or maintenance of IPCs.

To determine the cell cycle exit and proliferation indices in dcKO and control embryos at E15.5, we performed immunostaining for CIdU (24 h pulse) (marker of S-phase cells) and IdU (1 h pulse) in combination with Ki67 (marker for all active phases of the cell cycle) (Chenn and Walsh, 2002; Vega and Peterson, 2005) (Supplementary Figure S2A). The cell cycle exit index showed no significant difference between dcKO (rostral: 96%, caudal: 96%) and control (rostral: 95%, caudal: 96%) embryos. However, the proliferation index was reduced in dcKO cortices (rostral: 1%, caudal: 1%) compared to controls (rostral: 12.2%, caudal: 2.7%) (Supplementary Figures S2D,E), further supporting impaired production or maintenance of IPCs.

Together, these findings demonstrate that simultaneous loss of *Pbx1* and *Pbx3* selectively compromises the IPC pool, likely through impaired production or maintenance of these progenitors, while modestly altering RGC numbers.

Dual loss of Pbx1 and Pbx3 in cortex causes microcephaly and laminar defects with selective loss of upper layer neurons.

**FIGURE 3:**
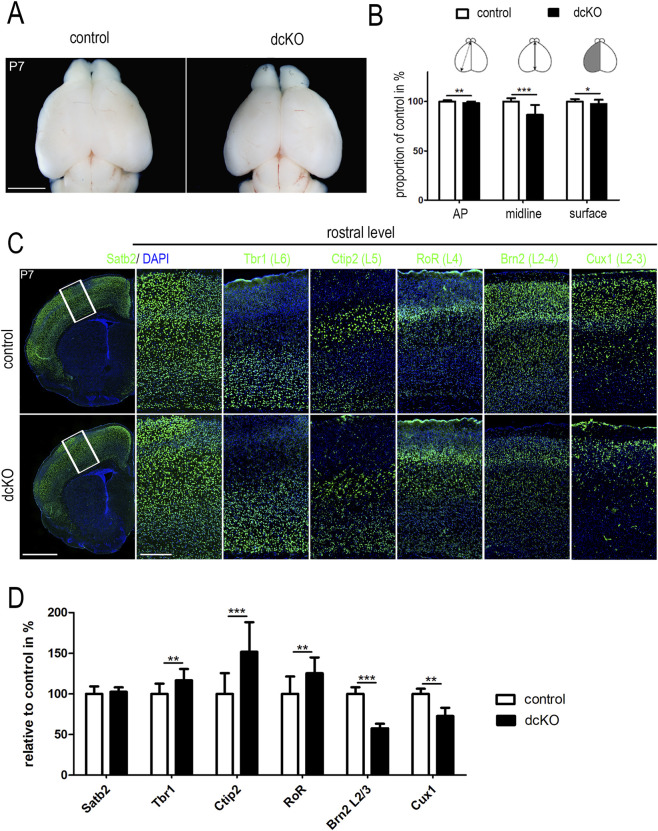
Loss of Pbx1 and Pbx3 causes microcephaly and leads to laminar defects. **(A)** At postnatal day 7 (P7), the cortex of dcKO mice was smaller than that of control littermates. **(B)** Quantification of cortical dimensions in dcKO mice relative to controls revealed significant reductions (AP: p = 1.8 × 10^−4^; midline: p = 3.59 × 10^−5^; surface: p = 0.031). **(C)** Immunohistochemical analysis of dcKO and control brains at P7, shown at rostral levels, using layer-specific markers: Satb2 (all layers), Tbr1 (layer 6), Ctip2 (layer 5), Rorb (layer 4), and Brn2 (layers 2–3). Scale bar: 1,000 µm. Higher-magnification images of boxed regions are shown on the right. Scale bar: 500 µm. **(D)** Quantification of layer-specific markers in dcKO cortices relative to controls at rostral levels. Data represents mean ± SD from three biological replicates, at least four images per replicate were used for quantification (n = 3, 12). Cell counts were obtained using CellProfiler. Statistical analysis was performed with Mann-Whitney-U test: p < 0.05, **p < 0.01, ***p < 0.001. Abbreviation: AP, anterior–posterior.

At postnatal day 7 (P7), dcKO cortices were noticeably smaller than those of control littermates (Figure 3A). Quantitative morphometric analysis confirmed a significant reduction in anterior-posterior length and midline thickness, resulting in an overall smaller cortical surface area, indicative of microcephaly (Figure 3B).

To determine whether cortical lamination was affected, we performed IHC analysis on coronal brain sections from dcKO and control mice at P7 using layer-specific neuronal molecular markers. These analyses revealed pronounced lamination defects in dcKO cortices at both rostral (Figures 3C,D) and caudal levels (Supplementary Figures S3A,B).

IHC for Satb2, which labels the majority of projection neurons across cortical layers, showed an overall unchanged population of Satb2^+^ neurons, with a more pronounced decrease in caudal regions (Figures 3C,D; Supplementary Figures S3A,B).

Further analysis of deep layer markers in rostral somatosensory areas at the level of the striatum and the anterior commissure (corresponding to image 50 of the Allen Brain Atlas, mouse, P56, coronal) revealed an increase in Tbr1^+^ cell number (layer (L) 6 marker) in dcKO cortices (Figures 3C,D). Tbr1^+^ cells were especially dense and clustered within the subplate (L6b) of dcKO cortices compared to controls (Figure 3C). In caudal somatosensory areas near the hippocampal formation (HPF) (corresponding to image 70 of the Allen Brain Atlas, mouse, P56, coronal), Tbr1 showed a similar increase in cell numbers (Supplementary Figures S3A,B).

IHC analysis for Ctip2, a molecular marker for L5 neurons and a regulator of callosal and subcerebral projections (Chen et al., 2008; Rodríguez-Tornos et al., 2016) indicated an overall increase in Ctip2^+^ cell numbers in dcKO cortices at both rostral and caudal levels (Figures 3C,D; Supplementary Figures S3A,B). At rostral levels, Ctip2^+^ neurons in dcKO brains lacked a distinct L5 band and were distributed across L5/6 (Figure 3C). In contrast, at caudal levels, the boundary between L5/6 was preserved, but the number of Ctip2^+^ neurons within L6 was increased Supplementary Figures S3A,B).

Analysis for *Rorb*, a marker for L4 neurons, revealed an expanded and thickened L4 in dcKO cortices compared to controls. While *Rorb* expression in controls was confined to a discrete L4, expression in dcKO cortices extended into L5 and was upregulated in L6 (Figures 3C,D; Supplementary Figures S3A,B). This phenotype was more pronounced in rostral regions. Collectively, these analyses demonstrate an overall expansion and misallocation of deep-layer neuronal identities in dcKO cortices along the rostro-caudal axis (Figures 3C,D).

In contrast to the increased numbers of deep-layer neurons, upper-layer neurons were significantly reduced in dcKO cortices. IHC and quantitative analyses of Brn2^+^ and Cux1^+^ upper layer (L2/3) neurons revealed a significant reduction in dcKO cortices at both rostral and caudal levels (Figures 3C,D; Supplementary Figures S3A,B). Total quantification of Brn2^+^ neurons showed a mild overall decrease across L2-4, with a significant reduction specifically in L2/3 in dcKO brains in comparison to control littermates (Figures 3C,D; Supplementary Figures S3A,B). Similarly, Cux1, a marker for layer 2/3 callosal projection neurons (Rodríguez-Tornos et al., 2016), was significantly reduced at both rostral and caudal levels in dcKO compared to controls (Figures 3C,D; Supplementary Figures S3A,B).

This rostro-caudal gradient in phenotype severity is consistent with the known rostro-caudal activity gradient of the *Emx1-*Cre driver (Lim et al., 2015; Kobeissy et al., 2016).

Together, these data demonstrate that loss of Pbx1 and Pbx3 reduces cortical size, disrupts laminar organization, alters deep-layer neuronal organization, and selectively depletes upper-layer neurons, indicating that Pbx1 and Pbx3 act cooperatively to maintain proper cortical development.

### 2.3 Deletion of Pbx1 and Pbx3 in forebrain leads to loss of the anterior commissure and partial agenesis of corpus callosum

**FIGURE 4:**
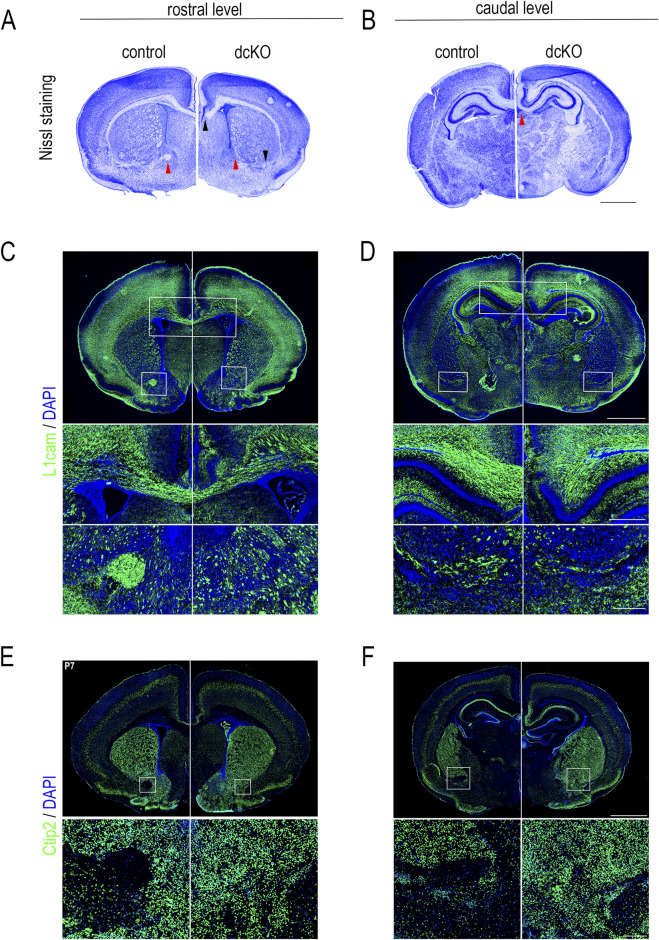
Dual deletion of Pbx1 and Pbx3 leads to a complete loss of the anterior commissure. **(A,B)** Cresyl violet–stained (Nissl) coronal brain sections from control and dcKO mice at rostral and caudal levels at postnatal day (P) 7. **(A)** The anterior commissure was completely absent in dcKO mice (upward red arrowhead), while its lateral portion appeared thinner and less defined than in controls (downward black arrowhead). **(B)** dcKO mice displayed partial agenesis of the corpus callosum (upward red arrowhead). Scale bar: 500 µm **(C–F)** Immunohistochemical analysis using Ctip2 and L1cam at rostral and caudal levels of dcKO and control brains at P7, reveals disrupted interhemispheric connectivity and partial agenesis of the corpus callosum in dcKO brains. **(C,E)** At rostral levels, dcKO brains exhibit a loss of the anterior commissure. **(D,F)** At caudal levels, the posterior branch of the anterior commissure appears thinned, shown at low (scale bar: 500 µm) and higher magnification (scale bar: 200 µm).

To examine cortical morphology and midline commissural structures, we performed a cresyl violet (Nissl) staining on coronal brain sections from *Pbx1*/*Pbx3*-dcKO and control littermates at P7 (Figures 4A,B; Supplementary Figure S4A).

At rostral levels (corresponding to image 40 of the Allen Brain Atlas, mouse, P56, coronal), dcKO brains exhibited markedly enlarged lateral ventricles (Supplementary Figure S4A, upward red arrowhead) and an abnormally dense ventricular zone (Supplementary Figure S4A, VZ; downward red arrowhead) compared with controls. In addition, the forceps anterior of the corpus callosum (CC) appeared thickened in dcKO mice (Supplementary Figure S4A, upward black arrowhead). At more caudal levels (corresponding to image 50 of the Allen Brain Atlas, mouse, P56, coronal), the anterior commissure (AC) was completely absent in dcKO mice (Figure 4A, upward red arrowhead), whereas it was clearly visible in control brains. Moreover, the lateral component of the AC in dcKO brains appeared thinner and poorly defined relative to controls (Figure 4A, downward black arrowhead).

Analysis along the rostro-caudal axis revealed partial CC defects in dcKO brains. At rostral sections, the CC was present but failed to cross the midline, in contrast to control littermates (Figure 4B, black upward arrowhead). At more posterior levels (corresponding to image 61 of the Allen Brain Atlas, mouse, P56, coronal), callosal fibers in dcKO mice were able to cross the midline, as observed in controls (Supplementary Figure S4B). However, the CC terminated prematurely (Figure 4B; Supplementary Figure S4C). Specifically, in dcKO brains the CC ended at a more anterior position, approximately at the level of the hippocampal formation (corresponding to image 70 of the Allen Brain Atlas), whereas in controls it extended further posteriorly (Figure 4B, upward red arrowhead; Supplementary Figure S4C).

Together, the Nissl staining data reveal pronounced structural defects in dcKO brains, including loss of the anterior commissure and partial agenesis of the corpus callosum.

To further assess interhemispheric connectivity, we performed immunohistochemical analysis of *L1cam* expression, an axon guidance and fasciculation molecule (Linneberg et al., 2019; Samata et al., 2020). At rostral levels, L1cam labeling confirmed thinning of the CC and absence of the AC in dcKO brains (Figure 4C). At caudal levels, sections were cut at a slight angle to capture both the posterior AC and callosal fibers in the same plane. Under these conditions, L1cam staining revealed premature termination of the callosal fibers at the level of the hippocampal formation in dcKO mice, in contrast to the continuous posterior extension observed in controls (Figure 4D, higher magnification). Consistent with these defects, immunostaining for the corticofugal neuron marker Ctip2 further revealed dysgenesis of commissural structures in dcKO mice, as indicated by the disrupted axonal organization highlighted by a white box (Figures 4E,F).

Together, these findings demonstrate that Pbx1 and Pbx3 are required for proper formation and organization of interhemispheric axonal connections. Their loss results in severe defects of the anterior commissure, midline integrity, callosal architecture, and organization of cortical projection neurons.

### 2.4 Pbx1 and Pbx3 regulate transcriptional programs controlling IPC lineage progression

**FIGURE 5:**
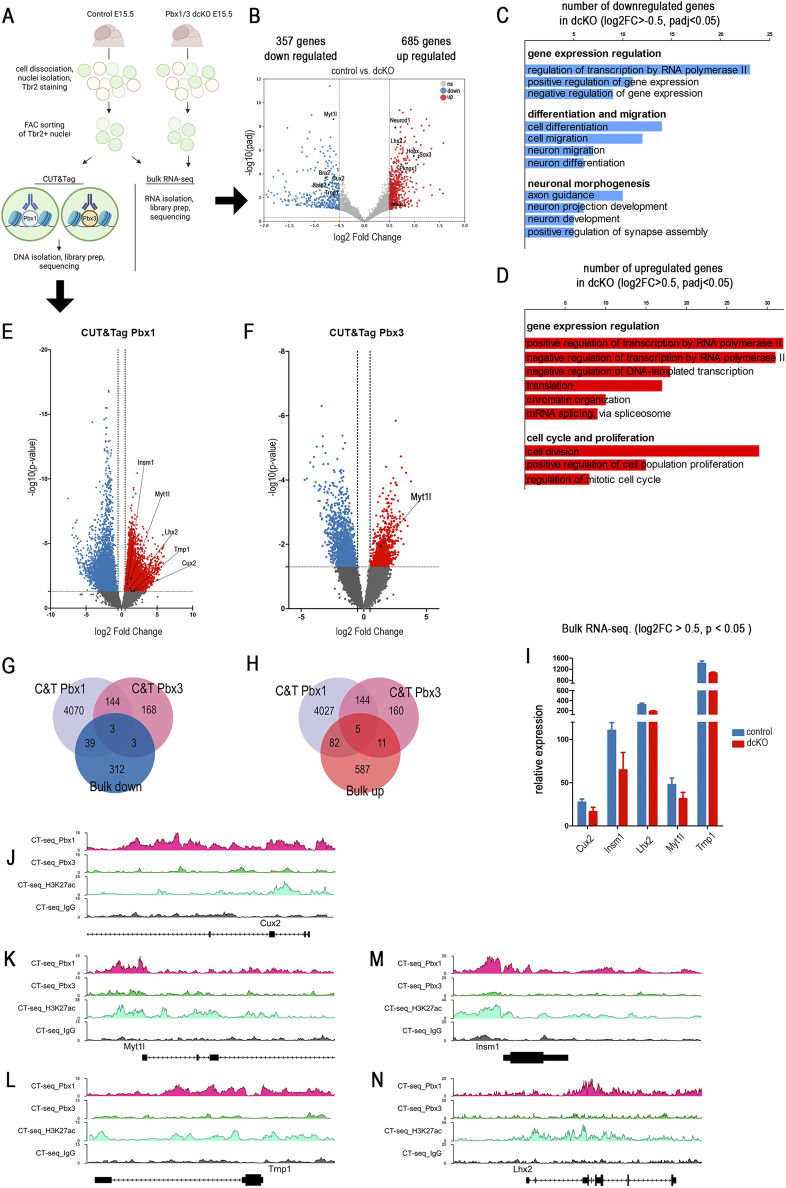
Integrated bulk RNA-seq and CUT&Tag analysis reveals novel Pbx1/Pbx3 targets. **(A)** Schematic overview for sample preparation for bulk-RNA-seq and CUT&Tag. **(B)** Volcano plot showing significant changes in gene expression revealed by bulk-RNA-seq from Tbr2^+^ IPCs of dcKO compared to controls at E15.5 (DESeq2, log2FC > 0.5, padj <0.05). **(C,D)** Gene ontology (GO) analysis of up- and downregulated transcripts identified by RNA-seq. **(E,F)** Volcano plots from CUT&Tag Pbx1 and CUT&Tag Pbx3 (DESeq2, log2FC > 0.5, p < 0.05) of control Tbr2^+^ IPCs showing differentially expressed genes. **(G,H)** Overlap between up- or downregulated genes from bulk-RNA-seq with Pbx1-or Pbx3-bound transcripts identified by CUT&Tag in E15.5 control mice. **(I)** Bar graph depicts differential expression of candidate genes from bulk-RNA-seq. **(J–N)** CUT&Tag genome browser tracks (20 kbp) displaying newly identified Pbx1/Pbx3 targets Cux2, Insm1, Lhx2, Myt1l and Trnp1.

Given the reduction in IPC number and proliferative capacity observed upon combined loss of *Pbx1* and *Pbx3*, we next sought to define the transcriptional programs regulated by these factors in IPCs. To this end, we compared the transcriptomic profiles of Tbr2^+^ IPCs isolated from dcKO and control cortices at E15.5. Nuclei were extracted from cortices, stained intranuclearly for *Tbr2,* and purified by FACS. Bulk RNA-seq libraries were generated from sorted Tbr2^+^ IPC populations and sequenced (Figure 5A).

Consistent with the profound IPC defects observed at the cellular level, differential expression analysis revealed widespread transcriptional alterations following *Pbx1*/*Pbx3* loss. In total, 1,042 genes were significantly dysregulated in dcKO IPCs, including 357 downregulated and 685 upregulated (DESeq2; |log_2_FC| > 0.5, padj <0.05; Figure 5B; Supplementary Figures S5A,B; Supplementary Table S1). Functional annotation of downregulated genes revealed strong enrichment for neuronal differentiation-associated categories, including regulation of gene expression, neuronal differentiation, neuronal morphogenesis, and axon guidance (Figure 5C). In contrast, genes upregulated in IPCs were predominately associated with regulation of gene expression, and cell cycle and proliferation (Figure 5D). These transcriptional signatures are broadly consistent with altered progenitor dynamics and disrupted IPC lineage progression in Pbx1/Pbx3-deficient cortices. Although cell cycle and proliferation genes were upregulated, this does not contradict the observed reduction in mitotic activity, as it likely reflects compensatory or stress-associated transcriptional responses in the remaining Tbr2^+^ population (Sessa et al., 2017; Zurkirchen et al., 2019).

To determine whether these transcriptional changes reflect direct regulation by *Pbx1* and *Pbx3*, we next mapped their chromatin occupancy in IPCs. Cleaved Under Targets and Tagmentation sequencing (CUT&Tag-seq) was performed on FACS-purified Tbr2^+^ IPC nuclei isolated from E15.5 cortices using antibodies against either Pbx1 or Pbx3. This analysis identified 4,256 Pbx1-bound genomic regions and 318 Pbx3-bound regions significantly enriched over IgG controls (DESeq2; log_2_FC > 0.5, p < 0.05; Figures 5E,F; Supplementary Figure S6; Supplementary Table S2), indicating that *Pbx1* exhibits widespread chromatin association, whereas *Pbx3* binds a more restricted subset of loci. These binding patterns were consistent with our snRNA-seq data, where Pbx1 is broadly expressed across IPC states, whereas Pbx3 is selectively enriched in proliferative IPCs (pIPC cluster; p < 0.001, log2FC = 1.67). This more restricted expression pattern is consistent with a reduced number of detectable binding events.

Integration of the bulk RNA-seq and CUT&Tag datasets allowed us to identify candidate direct transcriptional targets of Pbx1 and Pbx3. This approach revealed five upregulated and three downregulated genes that were both differentially expressed in dcKO IPCs and associated with Pbx1 and/or Pbx3 occupancy (Figures 5G,H). Notably, several of these shared targets encode regulators with well-established roles in IPC identity and lineage progression.

Among the downregulated candidate direct targets were *Cux2*, *Myt1l* and *Trnp1*, all of which have been implicated in promoting IPC proliferatison and neuronal fate acquisition (Cubelos et al., 2008; Stahl et al., 2013; Mall et al., 2017). Conversely, the upregulated candidate direct targets *Lhx2* and *Insm1* are associated with progenitor maintenance and neurogenic output (Farkas et al., 2008; Chou and O’Leary, 2013; Hsu et al., 2015). Differential expression of these candidates was confirmed by bulk-RNA-seq (Figure 5I). CUT&Tag profiling for Pbx1 demonstrated *Cux2*, *Insm1, Lhx2*, *Myt1l* and *Trnp1* occupancy at their regulatory regions, frequently overlapping with active chromatin marked by H3K27ac (Figures 5J–N). Pbx3 also occupied the *Myt1l* regulatory region, overlapping with H3K27ac-marked active chromatin (Figure 5K).

To determine whether *Pbx1/Pbx3* loss preferentially affects a specific IPC state, we integrated our bulk RNA-seq data with snRNA-seq-defined IPC signatures using CIBERSORTx deconvolution (Supplementary Figure S5C). This analysis did not reveal selective depletion of any individual IPC subpopulation, including pIPCs, suggesting that transcriptional dysregulation occurs broadly across IPC states rather than being confined to a single progenitor population.

Together, these data demonstrate that Pbx1 and Pbx3 regulate a core set of IPC genes involved in neuronal differentiation and progenitor state control. These findings support a model in which Pbx1 and Pbx3 orchestrate IPC genesis and maturation by directly modulating transcriptional programs governing IPC proliferation and lineage progression.

## 3 Discussion

Here, we investigated the roles of the transcription factors Pbx1 and Pbx3 in forebrain development using a loss-of-function approach. Our findings demonstrate that combined deletion of Pbx1 and Pbx3 in the developing forebrain results in a reduced pool of intermediate progenitor cells (IPCs), leading to cortical laminar disorganization and impaired interhemispheric connectivity. These abnormalities are accompanied by disruption of commissural and midline structures, including complete loss of the anterior commissure and partial agenesis of the corpus callosum (CC).

Members of the Pbx family, including Pbx1 and Pbx3, have previously been identified as critical regulators of forebrain development (Golonzhka et al., 2015; Grebbin et al., 2016; Hau et al., 2021). Consistent with earlier studies (Villaescusa et al., 2016; Laub et al., 2024), both Pbx1 and Pbx3 proteins remained detectable at E15.5 in *Emx1*-Cre-driven single conditional knockout (scKO) cortices, whereas both proteins were absent in double conditional knockout (dcKO) embryos (Supplementary Figures S1A–C). In addition, neither *Pbx1*-scKO nor *Pbx3*-scKO embryos showed detectable alterations in Tbr2^+^ cell numbers (Supplementary Figures S1D–F). Together, these findings are compatible with functional overlap between these highly homologous paralogs, although potential compensatory effects remain to be investigated. All subsequent analyses therefore focused on double conditional knockout (dcKO) mutants.

Histological analyses of *Pbx1/Pbx3*-dcKO mice revealed an overall reduction in the Tbr2^+^ IPC pool, accompanied by fewer Tbr2^+^/pHH3^+^ mitotic IPCs and an increased number of Pax6^+^/pHH3^+^ mitotic radial glial cells (RGCs). A significant reduction in the proliferation index was observed in the dcKO telencephalon, while cell cycle exit remained unchanged compared to controls. This imbalance between IPC and RGC mitotic activity is consistent with altered progenitor dynamics and impaired IPC generation and/or maintenance.

Transcriptomic analyses revealed broad dysregulation of genes associated with neurogenesis, cell cycle progression, and neuronal differentiation, consistent with altered progenitor dynamics and disrupted IPC lineage progression in *Pbx1/Pbx3*-deficient cortices. The upregulation of cell cycle-associated genes identified by GO analysis may reflect compensatory or stress-associated transcriptional responses in the residual Tbr2^+^ population that are nonetheless insufficient to restore normal upper-layer neurogenesis (Chenn and Walsh, 2002; Sessa et al., 2017; Nguyen et al., 2018).

Our CUT&Tag-seq datasets identified 4,256 binding peaks for Pbx1 and 318 for Pbx3 in Tbr2^+^ sorted IPCs. This more restricted binding pattern of Pbx3 is consistent with our snRNA-seq data, in which Pbx3 is selectively enriched in proliferative IPCs (pIPC cluster; p < 0.001, log2FC = 1.67), whereas Pbx1 is broadly expressed across all IPC states. Immunohistochemical analysis further supports lower and more spatially restricted Pbx3 expression relative to Pbx1. Together, these data suggest that the observed difference in peak numbers is likely to reflect genuine differences in chromatin occupancy rather than technical artifacts.

Integration of bulk RNA-seq and CUT&Tag-seq datasets identified a core set of candidate direct transcriptional targets associated with progenitor identity and IPC lineage progression, including Cux2, Insm1, Lhx2, Myt1l, and Trnp1. Trnp1 is a central regulator of RG identity that normally maintains apical progenitors while restricting premature basal progenitor formation (Volpe et al., 2006; Stahl et al., 2013). Its downregulation in dcKO IPCs suggests altered RGC-associated transcriptional programs and impaired progenitor progression. In parallel, Insm1 and Lhx2, regulators of progenitor identity and delamination (Farkas et al., 2008; Chou and O’Leary, 2013; Hsu et al., 2015), were upregulated following *Pbx1/Pbx3* loss, whereas reduced Myt1l expression indicates impaired neuronal differentiation (Mall et al., 2017). Cux2 marks fate-restricted RGC/IPC lineages that generate upper-layer callosal neurons, and its downregulation is consistent with the selective loss of upper-layer neurons and callosal defects observed postnatally (Cubelos et al., 2008; 2010; Franco et al., 2012).

Together, these findings suggest that *Pbx1/Pbx3* regulate multiple stages of IPC lineage progression and neuronal differentiation, potentially linking early progenitor progression with IPC- and neuron-associated transcriptional programs. Loss of *Pbx1/Pbx3* is associated with altered expression of genes linked to progenitor maintenance (*Trnp1, Lhx2, Insm1*), neuronal differentiation (*Myt1l*), and upper-layer neuron specification (*Cux2*), supporting disruption of a broader transcriptional network underlying cortical development (Cubelos et al., 2008; 2010; Farkas et al., 2008; Chou and O’Leary, 2013; Stahl et al., 2013; Mall et al., 2017).

At P7, dcKO cortices displayed pronounced laminar abnormalities accompanied by severe commissural defects. Similar phenotypes have been reported for transcription factor mutants affecting corticocortical connectivity, such as *Zbtb20* and *Satb2* (Nielsen et al., 2007; Rosenthal et al., 2012; Leone et al., 2015; Zhang et al., 2020). Mice lacking Zbtb20 or Satb2 display hippocampal malformations characterized by a curved morphology resembling that seen in dcKO mice (Supplementary Figure S4C) (Rosenthal et al., 2012; Zhang et al., 2020). Similarly, disruption of Cux1 (Rodríguez-Tornos et al., 2016) results in disrupted upper-layer neuron formation and CC hypoplasia, closely resembling the *Pbx1/Pbx3*-dcKO phenotype.

In particular, the upregulation of *Ctip2* (*Bcl11b*)*,* and altered *Satb2* expression in dcKO cortices parallel previous observations that *Satb2* loss promotes Ctip2 expression and altered specification of callosal *versus* corticofugal projection neuron identity (Leone et al., 2015; Paolino et al., 2020). Widespread Ctip2 misexpression across the anterior-posterior axis, combined with Satb2 repression specifically in posterior cortical regions, suggests a shared downstream regulatory pathway with *Satb2*-deficient mice, in which Ctip2 upregulation leads to malformed retrosplenial and subicular regions with densely clustered neurons (Supplementary Figures S4C,D) (Zhang et al., 2020).

These parallels support a model in which *Pbx1/Pbx3* activity contributes to the balance between distinct projection neuron lineages, potentially through Satb2-and Ctip2-associated transcriptional networks. Adequate repression of Ctip2 is crucial to preserve callosal projection neuron identity, while Ctip2 overexpression converts callosal neurons into subcerebral projection fates (Leone et al., 2015; Paolino et al., 2020). Whether the disorganization of interhemispheric axonal tracts and callosal misalignment arises from primary or secondary effects cannot be resolved in the present study.

Future studies employing lipophilic dye tracing, axonal labeling or AAV-based anterograde tracing in rostral and caudal cortical regions will be essential to determine whether cell-autonomous axonal guidance defects, or non-cell-autonomous effects arising from alterations in the cortical environment or midline structures, are the primary drivers of these phenotypes.

Notably, Pbx1/Pbx3 occupancy was detected at loci encoding *Tbr1*, *Ctip2*, *Rorb*, *Brn2*, *Cux1* and *L1cam*, suggesting association with transcriptional programs governing neuronal specification and axonal guidance (Supplementary Figure S7). Mutations in *L1cam* reduce callosal connectivity and cause axonal misrouting, pointing to convergent mechanisms between *Pbx1/Pbx3* regulated transcription and adhesion-mediated axon guidance (Yamasaki et al., 1997; Cheng and Lemmon, 2004; Shieh et al., 2015).

Overall, our data support a model in which *Pbx1* and *Pbx3* are required for proper laminar organization, interhemispheric connectivity, and CC formation. It remains an open question whether deep layer callosal neurons are directly affected by *Pbx1/Pbx3* deletion, and whether *Pbx1/Pbx3* act as transcriptional “pathfinders” for developing callosal projection neurons by integrating control of guidance cues with pioneer axon extension across the midline.

One possible explanation for the reduced IPC pool is altered neurogenic output from RGCs, potentially reflecting a shift from indirect toward more direct neurogenesis, which could prematurely deplete the progenitor pool and contribute to cortical disorganization. However, this possibility requires further experimental investigation, including lineage tracing. In addition, birth dating approaches combined with positional analyses will further help distinguish altered neuronal production from later migration or maturation defects.

Taken together, our data identify *Pbx1/Pbx3* as key transcriptional regulators of cortical development, linking progenitor dynamics with neuronal differentiation and forebrain connectivity. Combined loss of both Pbx paralogs in the developing telencephalon disrupts IPC-associated transcriptional programs, reduces the IPC pool, and results in cortical laminar abnormalities and commissural defects. These findings provide new insight into how coordinated transcriptional programs regulate forebrain morphogenesis and cortical organization.

## 4 Materials and methods

### 4.1 Transgenic mice

Floxed Pbx1 (B6.129S-*Pbx1* ^*tm3.1Mlc*^/J), floxed Pbx3 (B6; 129S4-*Pbx3* ^*tm1Og*^/J) and *Emx1*-Cre-mice (Gorski et al., 2002) mice were maintained in a C57BL6/J background. All animal experiments are ethically accomplished in agreement with the German Animal Welfare Act, the German Experimental Animals Ordinance and with the permission of LANUV North-Rhine-Westphalia.

### 4.2 Antibodies

The following polyclonal (pAb) and monoclonal (mAb) primary antibodies used in this study were obtained from the indicated commercial sources: Pbx1 rabbit pAb (1:200, Cell Signaling Technology, #4342S), Pbx3 rabbit pAb (1:200, Proteintech, 12571-1-AP), Pbx3 rabbit pAb (1:100, Sigma, AV32070), Satb2 mouse mAb (1:200, Abcam, ab51502), Tbr1 rabbit pAb (1:300, Abcam, ab31940), Ctip2 rat mAb (1:200, Abcam, ab18465), Rorb mouse mAb (1:100, Perseus Proteomics, PP-H3925-00), Brn2 goat pAb (1:100, Santa Cruz, sc- 6029), Cux1 rabbit pAb (1:200, Proteintech, 11733-1-AP), N-CAM L1 rat mAb (1:200, Chemicon, MAB5272), Sox2 rat mAb (1:400, eBioscience, 14-9811-82), Tuj1 mouse mAb (1:400, Chemicon, MAB1637), pHH3 rabbit pAb (1:400, Millipore, 06-570), CASP3 rabbit pAb (1:100; Cell Signaling, #9661S), PAX6 mouse mAb (1:100; Developmental Studies Hybridoma Bank, AB_528427), TBR2 rabbit pAb (1:200; Abcam, ab23345), TBR2 rat 923 mAb (1:200; eBioscience, 14-4875-82). Secondary antibodies used were Alexa 488-, Alexa 568-, Alexa 594- and Alexa 647- conjugated IgG (various species, 1:400; Molecular Probes).

### 4.3 Immunohistochemistry (IHC)

Immunohistochemistry (IHC) was performed as previously described (Tuoc et al., 2013; Narayanan et al., 2018). In brief, brain sections for IHC were blocked with normal sera of the appropriate species and incubated overnight at 4 °C with primary antibody. Primary antibodies were detected with a fluorescent secondary antibody of the respective species. Sections were counterstained with DAPI and mounted with ROTI®Mount FluorCare. Slides were dried overnight at room temperature.

### 4.5 Cresyl-violet staining

Brains of P7 dcKO and control littermates were isolated, cryo-embedded, sectioned at 16 μm, and stained with cresyl violet (Nissl) following standard procedures. Sections were mounted on SuperFrost Plus slides using DPX mounting medium.

### 4.6 Image acquisition and statistical analysis

Micrographs were obtained by confocal fluorescence microscopy (LSM800, LSM880, LSM980 Zeiss), widefield fluorescence microscope (Zeiss Axio Scan Z.1) and analyzed using ZenBlue 3.13 (Zeiss). Images were processed further using Fiji and Adobe Photoshop. The statistical quantification was carried out as average from at least three biological replicates, if not indicated. For statistical analysis of immunohistological staining a non-parametric Mann-Whitney-U test was performed. Detailed statistical analyses and descriptions of histological experiments are presented in Supplementary Table S3.

### 4.7 Bulk RNA-sequencing and bioinformatics analysis

RNA was obtained from cortices of ten control and ten dcKO embryos at E15.5. Cortices were dissociated and nuclei were stained intranuclear using Tbr2 antibody (rabbit pAb 1:200; Abcam) (Sakib et al., 2021; Ulmke et al., 2021). Nuclei were sorted using FACS. Sorted nuclei were collected in tubes pre-coated to minimize non-specific binding, then pelleted by brief centrifugation. RNA was extracted using TRIzol LS (Invitrogen) protocol, followed by aqueous phase purification with the Zymo RNA Clean and Concentrator-5 kit. RNA-seq libraries were generated using 1 ng of total RNA and the Takara SMART-Seq v4 Ultra Low Input RNA kit, following the manufacturer’s instructions. Libraries were split into four technical replicates and a paired-end Illumina sequencing was performed on the barcoded libraries using an Illumina NextSeq1000. Raw paired-end FASTQ files were processed in R (v4. x) using Bioconductor and associated packages. Sequencing read quality was assessed with FastQC (v0.12.1, http://www.bioinformatics.babraham.ac.uk/projects/fastqc/), and high quality reads were aligned to the *Mus musculus* reference genome (mm10, GRCm39 build) using Rsubread (v2.14.2) in RNA mode (Liao et al., 2019). Gene-level read counts were generated using featureCounts with GENCODE/UCSC annotation (gene_id). Differential expression analysis was performed using DESeq2 (v1.42.0) (Love et al., 2014). Genes with <10 counts in fewer than two samples were filtered out prior to model fitting. Size factors were estimated with estimateSizeFactors (), and differential expression was assessed using the Wald test with Benjamini–Hochberg false discovery rate (FDR) correction (α = 0.05). Log2 fold changes were shrunken using the apeglm method (Zhu et al., 2019). Quality control and visualization included principal component analysis (PCA) and sample-to-sample distance heatmaps using ggplot2 (v3.5.1) (Villanueva and Chen, 2019) and heatmap (v1.0.12) based on variance-stabilized counts from DESeq2. GO analyses were performed using the Database for Annotation, Visualization and Integrated Discovery (DAVID) (Huang, et al., 2009).

Deconvolution of bulk RNA-seq data was performed using the CIBERSORTx algorithm (Newman et al., 2015), which estimates the relative abundance of predefined cell types based on gene expression profiles. Normalized expression matrices from WT and *Pbx1/Pbx3*-dcKO bulk RNA-seq samples were used as input. A reference signature matrix was generated from single-nucleus RNA-seq data of mouse Tbr2^+^ cells. Deconvolution was performed to estimate the relative contribution of the Tbr2^+^ subpopulations within the bulk Tbr2-enriched populations.

### 4.8 Cleavage under targets and Tagmentation (CUT&TAG) sequencing and bioinformatics analysis

Libraries were generated from 16 wildtype CD1-mice cortices at stage E15.5. Cortices were homogenized, dissociated and intranuclearly stained using a Tbr2 rabbit pAb (1:75; Abcam), followed by FACS sorting (Sakib et al., 2021; Ulmke et al., 2021). CUT&Tag libraries were prepared using the CUT&Tag-IT™ Assay Kit–Tissue (Active Motif), according to the manufacturer’s instructions. For each sample 125,000 bound nuclei were used. Primary antibodies included Pbx1 rabbit pAb (1:100, Cell Signaling) and Pbx3 rabbit pAb (1:100, Sigma). Normal rabbit IgG (1:100, Cell Signaling Technology) served as negative control, and H3K27me3 rabbit pAb (1:100, Abcam) as positive control. Four technical replicates were prepared for each target, and a paired-end Illumina sequencing of barcoded libraries was performed on an Illumina NextSeq1000 platform. CUT&Tag sequencing data analysis followed the protocol of Zheng (2020). Peak calling was performed using MACS3 (version 3.0.2), and differential expression analysis was conducted using DESeq2 (version 1.42.1). No original code was developed for this study. Following gating and thresholding was used: baseMean >10, logFC >0.5, pvalue ≤0.05, annotation = promoter, distanceToTSS between −2000 and 2000. For visualization a custom Python script was used to intersect MACS3 peak regions with GENCODE vM30 gene annotations. Per-gene browser snapshots were created using pyGenomeTracks, and combined and individual plots were created with deepTools functions (computeMatrix, plotHeatmap, and plotProfile). Any additional information required to reanalyze the data reported in this paper is available from the lead contact upon request.

## Data Availability

The datasets presented in this study can be found in online repositories. This data can be found here: RNA-seq dataset: https://www.ncbi.nlm.nih.gov/sra/PRJNA1402735, CUT&Tag-seq dataset: https://www.ncbi.nlm.nih.gov/sra/PRJNA1414203, sn-RNA-seq dataset: https://www.ncbi.nlm.nih.gov/sra/PRJNA1469406.

## References

[B1] Chen B. Wang S. S. Hattox A. M. Rayburn H. Nelson S. B. McConnell S. K. (2008). The Fezf2-Ctip2 genetic pathway regulates the fate choice of subcortical projection neurons in the developing cerebral cortex. Proc. Natl. Acad. Sci. U. S. A. 105 (32), 11382–113827. 10.1073/pnas.0804918105 18678899 PMC2495013

[B2] Cheng L. Lemmon V. (2004). Pathological missense mutations of neural cell adhesion molecule L1 affect neurite outgrowth and branching on an L1 substrate. Mol. Cell. Neurosci. 27 (4), 522–530. 10.1016/j.mcn.2004.08.005 15555929

[B3] Chenn A. Walsh C. A. (2002). “Regulation of cerebral cortical size by control of cell cycle exit in neural precursors,” Science, 297, 365–369. 10.1126/science.1074192 12130776

[B4] Chou S. J. O’Leary D. D. M. (2013). Role for Lhx2 in corticogenesis through regulation of progenitor differentiation. Mol. Cell. Neurosci. 56, 1–9. 10.1016/j.mcn.2013.02.006 23454273 PMC3706495

[B5] Crisafulli L. Brindisi M. Liturri M. G. Sobacchi C. Ficara F. (2024). PBX1: a TALE of two seasons—key roles during development and in cancer. Front. Cell Dev. Biol. 12, 1372873. 10.3389/fcell.2024.1372873 38404687 PMC10884236

[B6] Cubelos B. Sebastián-Serrano A. Kim S. Moreno-Ortiz C. Redondo J. M. Walsh C. A. (2008). Cux-2 controls the proliferation of neuronal intermediate precursors of the cortical subventricular Zone. Cereb. Cortex 18 (8), 1758–1770. 10.1093/cercor/bhm199 18033766

[B7] Cubelos B. Sebastián-Serrano A. Beccari L. Calcagnotto M. E. Cisneros E. Kim S. (2010). Cux1 and Cux2 regulate dendritic branching, spine morphology, and synapses of the upper layer neurons of the cortex. Neuron 66 (4), 523–535. 10.1016/j.neuron.2010.04.038 20510857 PMC2894581

[B8] Farkas L. M. Haffner C. Giger T. Khaitovich P. Nowick K. Birchmeier C. (2008). Insulinoma-Associated 1 has a panneurogenic role and promotes the generation and expansion of basal progenitors in the developing mouse neocortex. Neuron 60 (1), 40–55. 10.1016/j.neuron.2008.09.020 18940587

[B9] Fitzgerald K. K. Powell-Hamilton N. Shillingford A. J. Robinson B. Gripp K. W. (2021). Inherited intragenic PBX1 deletion: expanding the phenotype. Am. J. Med. Genet. Part A 185 (1), 234–237. 10.1002/ajmg.a.61932 33098248

[B10] Franco S. J. Gil-Sanz C. Martinez-Garay I. Espinosa A. Harkins-Perry S. R. Ramos C. (2012). Fate-Restricted neuronal progenitors in the mammalian cerebral cortex. Science 337 (6095), 746–749. 10.1126/science.1223616 22879516 PMC4287277

[B11] Gavrish M. Kustova A. Celis Suescún J. C. Bessa P. Mitina N. Tarabykin V. (2023). Molecular mechanisms of corpus callosum development: a four-step journey, in Front. Neuroanat. 17 1276325. 10.3389/fnana.2023.1276325 38298831 PMC10827913

[B12] Golonzhka O. Nord A. Tang P. L. F. Lindtner S. Ypsilanti A. R. Ferretti E. (2015). Pbx regulates patterning of the cerebral cortex in progenitors and postmitotic neurons. Neuron 88 (6), 1192–1207. 10.1016/j.neuron.2015.10.045 26671461 PMC4688141

[B13] Gorski J. A. Talley T. Qiu M. Puelles L. Rubenstein J. L. Jones K. R. (2002). Brief communication cortical excitatory neurons and glia, but not GABAergic neurons, are produced in the Emx1-Expressing lineage. Available online at: http://www.jneurosci.org (Accessed March 23, 2023). 10.1523/JNEUROSCI.22-15-06309.2002 PMC6758181 12151506

[B14] Götz M. Huttner W. B. (2005). The cell biology of neurogenesis. Nat. Rev. Mol. Cell Biol. 6, 777–788. 10.1038/nrm1739 16314867

[B15] Grebbin B. M. Schulte D. (2017). PBX1 as pioneer factor: a case still open. Front. Cell Dev. Biol. 5, 9. 10.3389/fcell.2017.00009 28261581 PMC5306212

[B16] Grebbin B. M. Hau A. C. Groß A. Anders-Maurer M. Schramm J. Koss M. (2016). PBX1 is required for adult subventricular zone neurogenesis. Dev. Camb. 143 (13), 2281–2291. 10.1242/dev.128033 27226325 PMC4958316

[B17] Hanley O. Zewdu R. Cohen L. J. Jung H. Lacombe J. Philippidou P. (2016). Parallel pbx-dependent pathways govern the coalescence and fate of motor columns. Neuron 91 (5), 1005–1020. 10.1016/j.neuron.2016.07.043 27568519 PMC5017921

[B18] Hau A. C. Mommaerts E. Laub V. Müller T. Dittmar G. Schulte D. (2021). Transcriptional cooperation of PBX1 and PAX6 in adult neural progenitor cells. Sci. Rep. 11 (1), 21013. 10.1038/s41598-021-99968-5 34697387 PMC8545929

[B19] Haubensak W. Attardo A. Denk W. Huttner W. B. Simons K. (2004). Neurons arise in the basal neuroepithelium of the early mammalian telencephalon: a major site of neurogenesis. PNAS March. 101, 3196–3201. 10.1073/pnas.0308600100 14963232 PMC365766

[B20] Hsu L. C. L. Nam S. Cui Y. Chang C. P. Wang C. F. Kuo H. C. (2015). Lhx2 regulates the timing of β-catenin-dependent cortical neurogenesis. Proc. Natl. Acad. Sci. U. S. A. 112 (39), 12199–12204. 10.1073/pnas.1507145112 26371318 PMC4593128

[B21] Huang D. W. Sherman B. T. Lempicki R. A. (2009). Systematic and integrative analysis of large gene lists using DAVID bioinformatics resources. Nat. Protoc. 4 (1), 44–57. 10.1038/nprot.2008.211 19131956

[B22] Kerimoglu C. Pham L. Tonchev A. B. Sakib M. S. Xie Y. Sokpor G. (2021). H3 acetylation selectively promotes basal progenitor proliferation and neocortex expansion. Sci. Adv. 7, 6792–6807. 10.1126/sciadv.abc6792 34524839 PMC8443185

[B23] Kobeissy F. H. Hansen K. Neumann M. Fu S. Jin K. Liu J. (2016). Deciphering the role of Emx1 in neurogenesis: a neuroproteomics approach. Front. Mol. Neurosci. 9 (OCT2016), 98. 10.3389/fnmol.2016.00098 27799894 PMC5065984

[B24] Kriegstein A. Alvarez-Buylla A. (2009). The glial nature of embryonic and adult neural stem cells. Annu. Rev. Neurosci. 32, 149–184. 10.1146/annurev.neuro.051508.135600 19555289 PMC3086722

[B25] Laub V. Nan E. Elias L. Donaldson I. J. Bentsen M. Rusling L. A. (2024). Integrated multi-omics analysis of PBX1 in mouse adult neural stem- and progenitor cells identifies a transcriptional module that functionally links PBX1 to TCF3/4. Nucleic Acids Res. 52 (20), 12262–12280. 10.1093/nar/gkae864 39377397 PMC11551771

[B26] Leone D. P. Heavner W. E. Ferenczi E. A. Dobreva G. Huguenard J. R. Grosschedl R. (2015). Satb2 regulates the differentiation of both callosal and subcerebral projection neurons in the developing cerebral cortex. Cereb. Cortex 25 (10), 3406–3419. 10.1093/cercor/bhu156 25037921 PMC4585495

[B27] Liao Y. Smyth G. K. Shi W. (2019). The R package Rsubread is easier, faster, cheaper and better for alignment and quantification of RNA sequencing reads. Nucleic Acids Res. 47 (8), e47. 10.1093/nar/gkz114 30783653 PMC6486549

[B28] Lim J. W. C. Donahoo A. L. S. Bunt J. Edwards T. J. Fenlon L. R. Liu Y. (2015). EMX1 regulates NRP1-mediated wiring of the mouse anterior cingulate cortex. Dev. Camb. 142 (21), 3746–3757. 10.1242/dev.119909 26534986 PMC4647209

[B29] Linneberg C. Toft C. L. F. Kjaer-Sorensen K. Laursen L. S. (2019). L1cam-mediated developmental processes of the nervous system are differentially regulated by proteolytic processing. Sci. Rep. 9 (1), 3716. 10.1038/s41598-019-39884-x 30842511 PMC6403279

[B30] Liu Y. Ao X. Zhuo X. Du C. Kuang S. (2022). “The regulation of PBXs and their emerging role in cancer,” in Journal of Cellular and Molecular Medicine. John Wiley and Sons Inc, 1363–1379. 10.1111/jcmm.17196 PMC8899182 35068042

[B31] Love M. I. Huber W. Anders S. (2014). Moderated estimation of fold change and dispersion for RNA-seq data with DESeq2. Genome Biol. 15 (12), 550. 10.1186/s13059-014-0550-8 25516281 PMC4302049

[B32] Mall M. Kareta M. S. Chanda S. Ahlenius H. Perotti N. Zhou B. (2017). Myt1l safeguards neuronal identity by actively repressing many non-neuronal fates. Nature 544 (7649), 245–249. 10.1038/nature21722 28379941 PMC11348803

[B33] Martínez-Cerdeño V. Noctor S. C. Kriegstein A. R. (2006). The role of intermediate progenitor cells in the evolutionary expansion of the cerebral cortex. Cereb. Cortex 16 (Suppl. 1), i152–i161. 10.1093/cercor/bhk017 16766701

[B34] Mihrshahi R. (2006). The corpus callosum as an evolutionary innovation. J. Exp. Zoology Part B Mol. Dev. Evol. 306, 8–17. 10.1002/jez.b.21067 16116611

[B35] Narayanan R. Pham L. Kerimoglu C. Watanabe T. Castro Hernandez R. Sokpor G. (2018). Chromatin remodeling BAF155 subunit regulates the genesis of basal progenitors in developing cortex. iScience 4, 109–126. 10.1016/j.isci.2018.05.014 30240734 PMC6147019

[B36] Newman A. M. Liu C. L. Green M. R. Gentles A. J. Feng W. Xu Y. (2015). Robust enumeration of cell subsets from tissue expression profiles. Nat. Methods 12 (5), 453–457. 10.1038/nmeth.3337 25822800 PMC4739640

[B37] Nguyen H. Kerimoglu C. Pirouz M. Pham L. Kiszka K. A. Sokpor G. (2018). Epigenetic regulation by BAF complexes limits neural stem cell proliferation by suppressing wnt signaling in late embryonic development. Stem Cell Rep. 10 (6), 1734–1750. 10.1016/j.stemcr.2018.04.014 29779894 PMC5993560

[B38] Nielsen J. V. Nielsen F. H. Ismail R. Noraberg J. Jensen N. A. (2007). Hippocampus-like corticoneurogenesis induced by two isoforms of the BTB-zinc finger gene Zbtb20 in mice. Development 134 (6), 1133–1140. 10.1242/dev.000265 17301088

[B39] Paolino A. Fenlon L. R. Kozulin P. Haines E. Lima J. W. C. Richards L. J. (2020). Differential timing of a conserved transcriptional network underlies divergent cortical projection routes across mammalian brain evolution. PNAS 117 (19), 10554–10564. 10.1073/pnas.1922422117/-/DCSupplemental 32312821 PMC7229759

[B40] Rhee J. W. Arata A. Selleri L. Jacobs Y. Arata S. Onimaru H. (2004). Pbx3 deficiency results in central hypoventilation. Am. J. Pathology 165 (4), 1343–1350. 10.1016/S0002-9440(10)63392-5 15466398 PMC1618620

[B41] Rodríguez-Tornos F. M. Briz C. G. Weiss L. A. Sebastián-Serrano A. Ares S. Navarrete M. (2016). Cux1 enables interhemispheric connections of layer II/III neurons by regulating Kv1-Dependent firing. Neuron 89 (3), 494–506. 10.1016/j.neuron.2015.12.020 26804994

[B42] Rosenthal E. H. Tonchev A. B. Stoykova A. Chowdhury K. (2012). Regulation of archicortical arealization by the transcription factor Zbtb20. Hippocampus 22 (11), 2144–2156. 10.1002/hipo.22035 22689450

[B43] Rottkamp C. A. Lobur K. J. Wladyka C. L. Lucky A. K. O'Gorman S. (2008). Pbx3 is required for normal locomotion and dorsal horn development. Dev. Biol. 314 (1), 23–39. 10.1016/j.ydbio.2007.10.046 18155191

[B44] Sakib M. S. Sokpor G. Nguyen H. P. Fischer A. Tuoc T. (2021). Intranuclear immunostaining-based FACS protocol from embryonic cortical tissue. Star. Protoc. 2 (1), 100318. 10.1016/j.xpro.2021.100318 33554149 PMC7859298

[B45] Samata B. Takaichi R. Ishii Y. Fukushima K. Nakagawa H. Ono Y. (2020). L1CAM is a marker for enriching corticospinal motor neurons in the developing brain. Front. Cell. Neurosci. 14, 31. 10.3389/fncel.2020.00031 32140099 PMC7042175

[B46] Selleri L. Zappavigna V. Ferretti E. (2019). “‘Building a perfect body’: control of vertebrate organogenesis by PBX-dependent regulatory networks.” 10.1101/gad.318774 PMC6411007 30824532

[B47] Sessa A. Ciabatti E. Drechsel D. Massimino L. Colasante G. Giannelli S. (2017). The Tbr2 molecular network controls cortical neuronal differentiation through complementary genetic and epigenetic pathways. Cereb. Cortex 27 (6), 3378–3396. 10.1093/cercor/bhw270 27600842

[B48] Shieh C. Moser F. Graham J. M. Watiker V. Pierson T. M. (2015). Mutation in the sixth immunoglobulin domain of L1CAM is associated with migrational brain anomalies. Neurol. Genet. 1 (4), e34. 10.1212/NXG.0000000000000034 27066571 PMC4811382

[B49] Slavotinek A. Risolino M. Losa M. Cho M. T. Monaghan K. G. Schneidman-Duhovny D. (2017). *De novo,* deleterious sequence variants that alter the transcriptional activity of the homeoprotein PBX1 are associated with intellectual disability and pleiotropic developmental defects. Hum. Mol. Genet. 26 (24), 4849–4860. 10.1093/hmg/ddx363 29036646 PMC6455034

[B50] Stahl R. Walcher T. De Juan Romero C. Pilz G. A. Cappello S. Irmler M. (2013). Trnp1 regulates expansion and folding of the mammalian cerebral cortex by control of radial glial fate. Cell 153 (3), 535–549. 10.1016/j.cell.2013.03.027 23622239

[B51] Tuoc T. C. Boretius S. Sansom S. N. Pitulescu M. E. Frahm J. Livesey F. J. (2013). Chromatin regulation by BAF170 Controls Cerebral Cortical Size and thickness. Dev. Cell 25 (3), 256–269. 10.1016/j.devcel.2013.04.005 23643363

[B52] Ulmke P. A. Sakib M. S. Ditte P. Sokpor G. Kerimoglu C. Pham L. (2021). Molecular profiling reveals involvement of ESCO2 in intermediate progenitor cell maintenance in the developing mouse cortex. Stem Cell Rep. 16 (4), 968–984. 10.1016/j.stemcr.2021.03.008 33798452 PMC8072132

[B53] Vega C. J. Peterson D. A. (2005). Stem cell proliferative history in tissue revealed by temporal halogenated thymidine analog discrimination. Nat. Methods 2 (3), 167–169. 10.1038/nmeth741 15782184

[B54] Villaescusa J. C. Li B. Toledo E. M. Rivetti di Val Cervo P. Yang S. Stott S. R. (2016). A PBX1 transcriptional network controls dopaminergic neuron development and is impaired in Parkinson’s disease. EMBO J. 35 (18), 1963–1978. 10.15252/embj.201593725 27354364 PMC5282836

[B55] Villanueva R. A. M. Chen Z. J. (2019). ggplot2: elegant graphics for data analysis. Meas. Interdiscip. Res. Perspect. 17 (3), 160–167. 10.1080/15366367.2019.1565254

[B56] Volpe M. Shpungin S. Barbi C. Abrham G. Malovani H. Wides R. (2006). Trnp: a conserved Mammalian gene encoding a nuclear protein that accelerates cell-cycle progression. DNA CELL Biol. 25, 331–339. 10.1089/dna.2006.25.331 16792503

[B57] Yamasaki M. Thompson P. Lemmon V. (1997). CRASH syndrome: mutations in L1CAM correlate with severity of the disease. Neuropediatrics 28, 175–178. 10.1055/s-2007-973696 9266556 PMC1563987

[B58] Zewdu R. Risolino M. Barbulescu A. Ramalingam P. Butler J. M. Selleri L. (2016). Spleen hypoplasia leads to abnormal stress hematopoiesis in mice with loss of Pbx homeoproteins in splenic mesenchyme. J. Anat. 229 (1), 153–169. 10.1111/joa.12479 27075259 PMC5341595

[B59] Zhang L. Song N. N. Zhang Q. Mei W. Y. He C. H. Ma P. (2020). Satb2 is required for the regionalization of retrosplenial cortex. Cell Death Differ. 27 (5), 1604–1617. 10.1038/s41418-019-0443-1 31666685 PMC7206047

[B60] Zhao Y. bai X. Li N. Zheng N. Si Y. Zhao Y. (2024). PBX3 as a biomarker for the early diagnosis and prediction of prognosis of glioma. PLoS ONE 19 (2 February), e0293647. 10.1371/journal.pone.0293647 38324550 PMC10849273

[B61] Zheng Y. (2020). CUT&Tag data processing analysis tutorial. GitHub. 10.17504/protocols.io.bjk2kkye

[B62] Zhu A. Ibrahim J. G. Love M. I. (2019). Heavy-tailed prior distributions for sequence count data: removing the noise and preserving large differences. Bioinformatics 35 (12), 2084–2092. 10.1093/bioinformatics/bty895 30395178 PMC6581436

[B63] Zurkirchen L. Varum S. Giger S. Klug A. Häusel J. Bossart R. (2019). Yin Yang 1 sustains biosynthetic demands during brain development in a stage-specific manner. Nat. Commun. 10 (1), 2192. 10.1038/s41467-019-09823-5 31097699 PMC6522535

