# Supplementary material for: Pbx1 and Pbx3 cooperatively regulate intermediate progenitor genesis and corticogenesis in the mouse neocortex
Source: Front Cell Dev Biol. 2026 Jul 2;14:1809251. doi: 10.3389/fcell.2026.1809251 (PMC13373412; doi:10.3389/fcell.2026.1809251)
Supplement: Supplementary file 1 [file Supplementaryfile1.docx]

Supplementary materials for

**Pbx1 and Pbx3 Cooperatively Regulate Intermediate Progenitor Genesis and Corticogenesis in the Mouse Neocortex**

Asisa Muchamedin^1^, Pauline A. Ulmke^1^, Linh Pham^1^, Hoang D. Nguyen^1^, Marie-Luise Kümmel^2^, Boris Burr^2^ David Bietz^3^, Petra Wahle^3^, Huu Phuc Nguyen^1^, and Tran Tuoc^1, *^

^1^Department of Human Genetics, Ruhr University of Bochum, Bochum, Germany

^2^ Department of Neuroanatomy, Ruhr University of Bochum, Bochum, Germany

^3^Department of Developmental Biology, Ruhr University of Bochum, Bochum, Germany

*** Correspondence:**Tran Tuoc
Tran.Tuoc@ruhr-uni-bochum.de

**This file includes:**

Supplementary Figures S1-S7 with legends

**
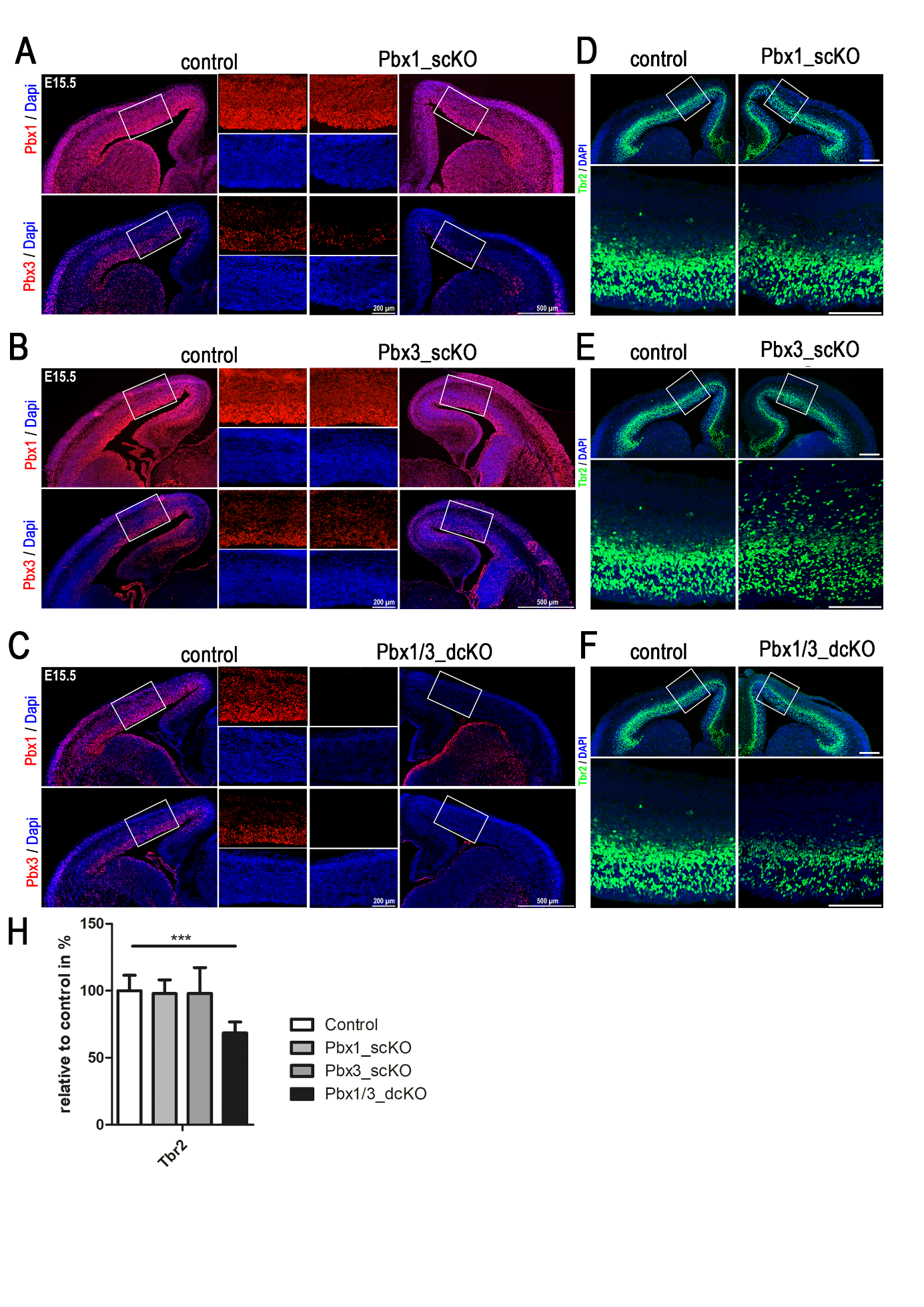
**

**Figure S1: Expression of Pbx1, Pbx3 and Tbr2 in the developing mouse cortex of single and double conditional knockouts mouse mutants.** (A-F) Immunohistochemistry showing cortical expression of Pbx1, Pbx3 and Tbr2 in coronal forebrain sections of control, Pbx1_scKO, Pbx3_scKO and Pbx1/3_dcKO at E15.5. (A, D) Pbx1 expression is preserved in Pbx1_scKO and no significant differences in the number of Tbr2^+^ cells were observed compared to controls. (B, E) Pbx3 expression is similarly retained in Pbx3_scKO, with no significant alterations in Tbr2^+^ cell numbers compared to controls. (C, F) In contrast, Pbx1/3_dcKO embryos exhibited a complete loss of both Pbx1 and Pbx3 expressions in the cortex, accompanied by a significant reduction in the number of Tbr2^+^ cells compared to controls. (H) Data represents mean ± SD from two biological replicates, at least 4 four images per replicate were used for quantification (n=2, 12). Cell counts were obtained using CellProfiler. Statistical analysis was performed with Mann-Whitney-U test: p < 0.05, **p < 0.01, ***p < 0.001. Pbx, pre-B-cell leukemia transcription factor; scKO, single conditional knockout; Tbr2, T-box transcription factor 2; dcKO, double conditional knockout;


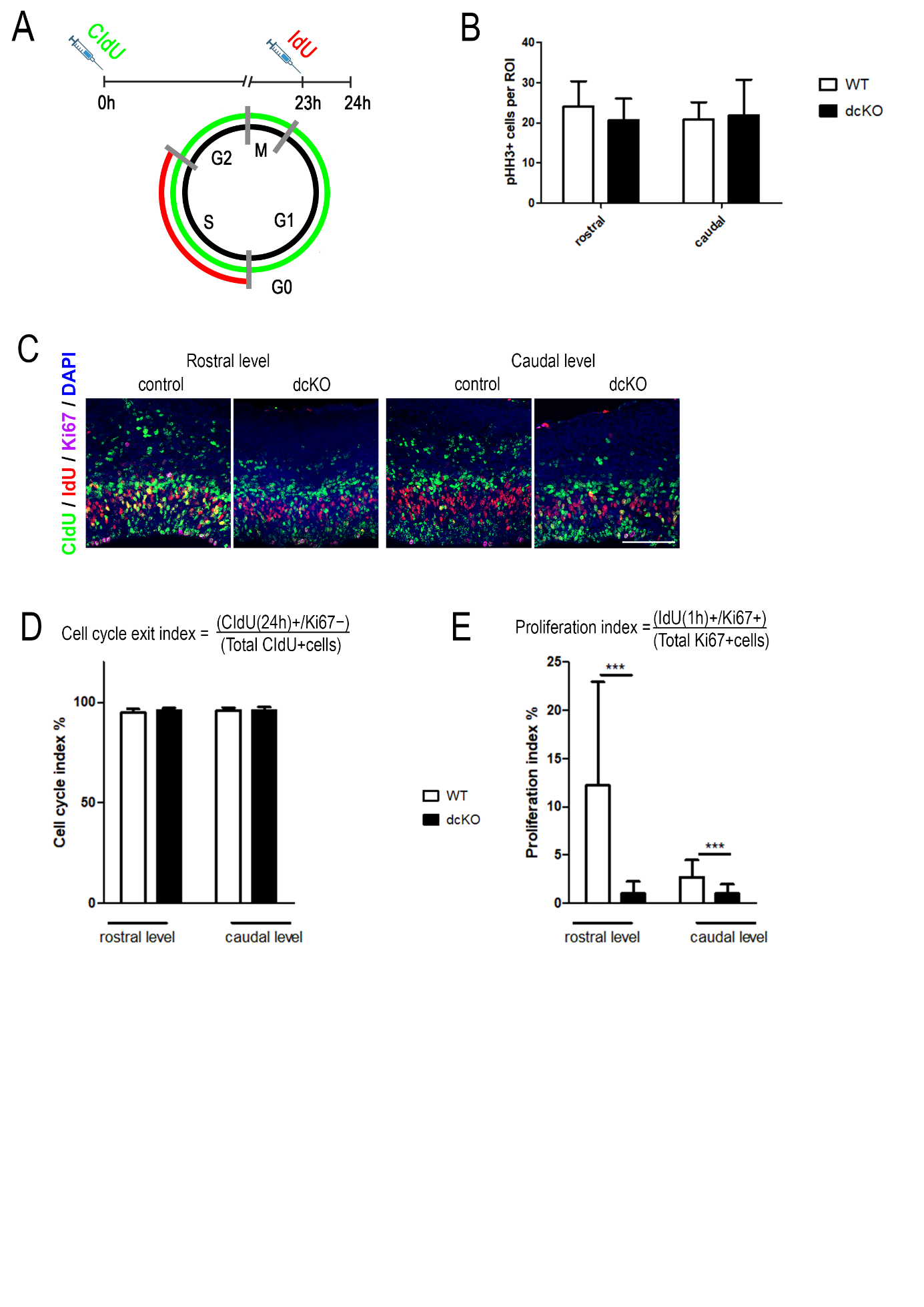


**Figure S2: Proliferation and mitotic activity in dcKO brains at E15.5.** (A) Schematic of nucleoside analog injection: CIdU was administered for 24 hours to label cells both in and exiting the cell cycle, and IdU for 1 hour to label progenitors in S-phase. (B) Quantification of total pHH3+ cells. The total number of pHH3+ cells did not differ between dcKO and control cortices. (C) Triple immunohistochemical staining for CIdU, IdU and Ki67 (a proliferation marker) in dcKO and control cortices. (D-E) Cell cycle exit and proliferation indices. Data represent mean ± SD from five biological replicates, at least 4 four images per replicates was used for quantification (n=5, 20). Quantification was performed using CellProfiler. Statistical analysis was performed with Mann-Whitney-U test: p < 0.05, **p < 0.01, ***p < 0.001. M, mitosis; S, synthesis; G1, gap1; G2, gap2; G0 resting phase; CIdU, chloro-deoxyuridine; IdU, iodo-deoxyuridine; dcKO, double conditional knockout;


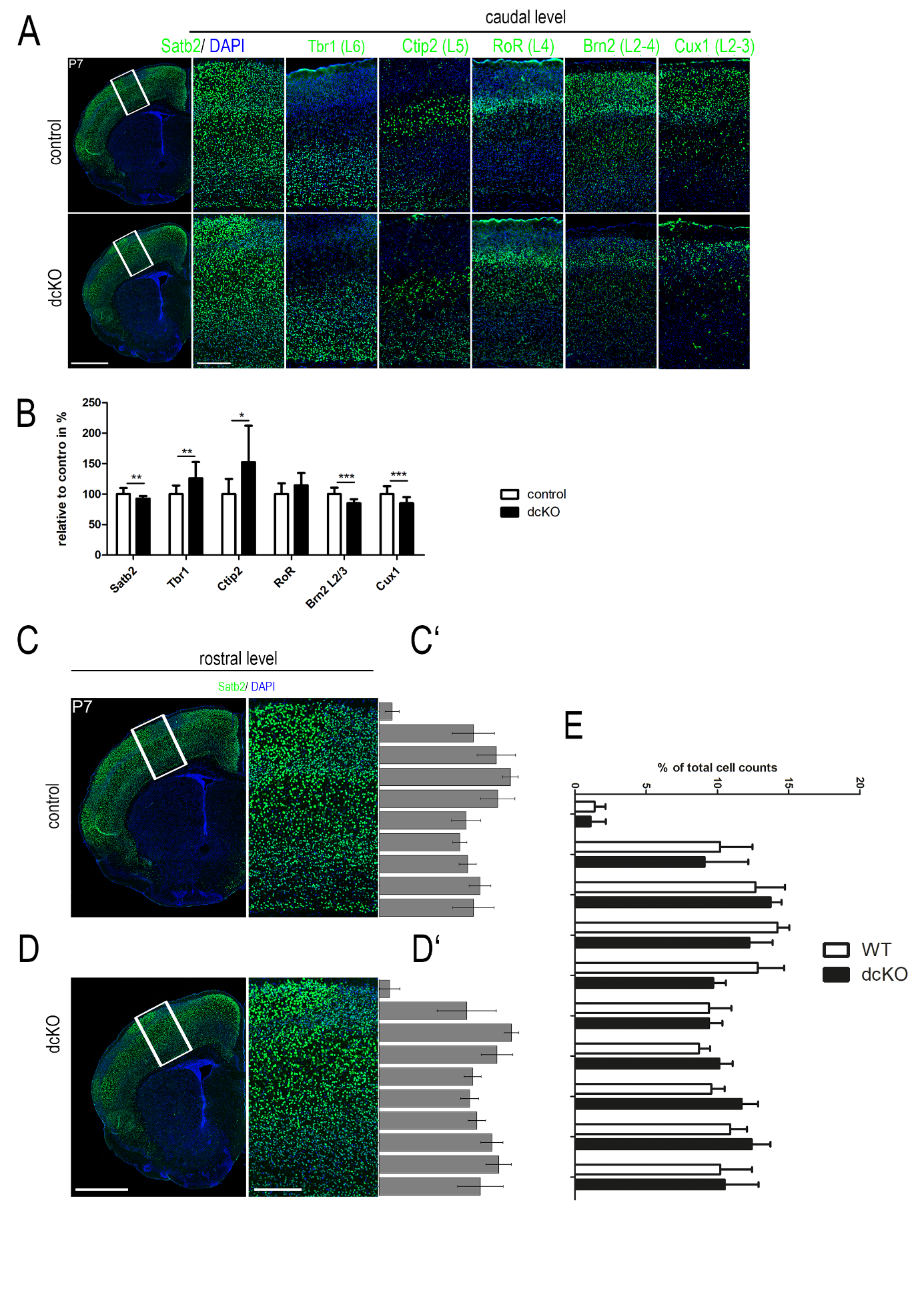


**Figure S3: Dual deletion of Pbx1 and Pbx3 leads to laminar defects at caudal levels.**
(A) Immunohistochemical analysis of dcKO and control brains at P7, shown at caudal levels, using layer-specific markers: Satb2 (all layers), Tbr1 (layer 6), Ctip2 (layer 5), Rorβ (layer 4), and Brn2 (layers 2–3). Scale bar: 1000 µm. Higher-magnification images of boxed regions are shown on the right. Scale bar: 500 µm. (B) Quantification of layer-specific markers in dcKO cortices relative to controls at caudal levels. (C-E) Similar distribution of Satb2^+^ cells along the radial axis of P7 control and dcKO cortex. Data represents mean ± SD from three biological replicates, at least 4 four images per replicate were used for quantification (n=3, 12). Cell counts were obtained using CellProfiler. Statistical analysis was performed with Mann-Whitney-U test: p < 0.05, **p < 0.01, ***p < 0.001.


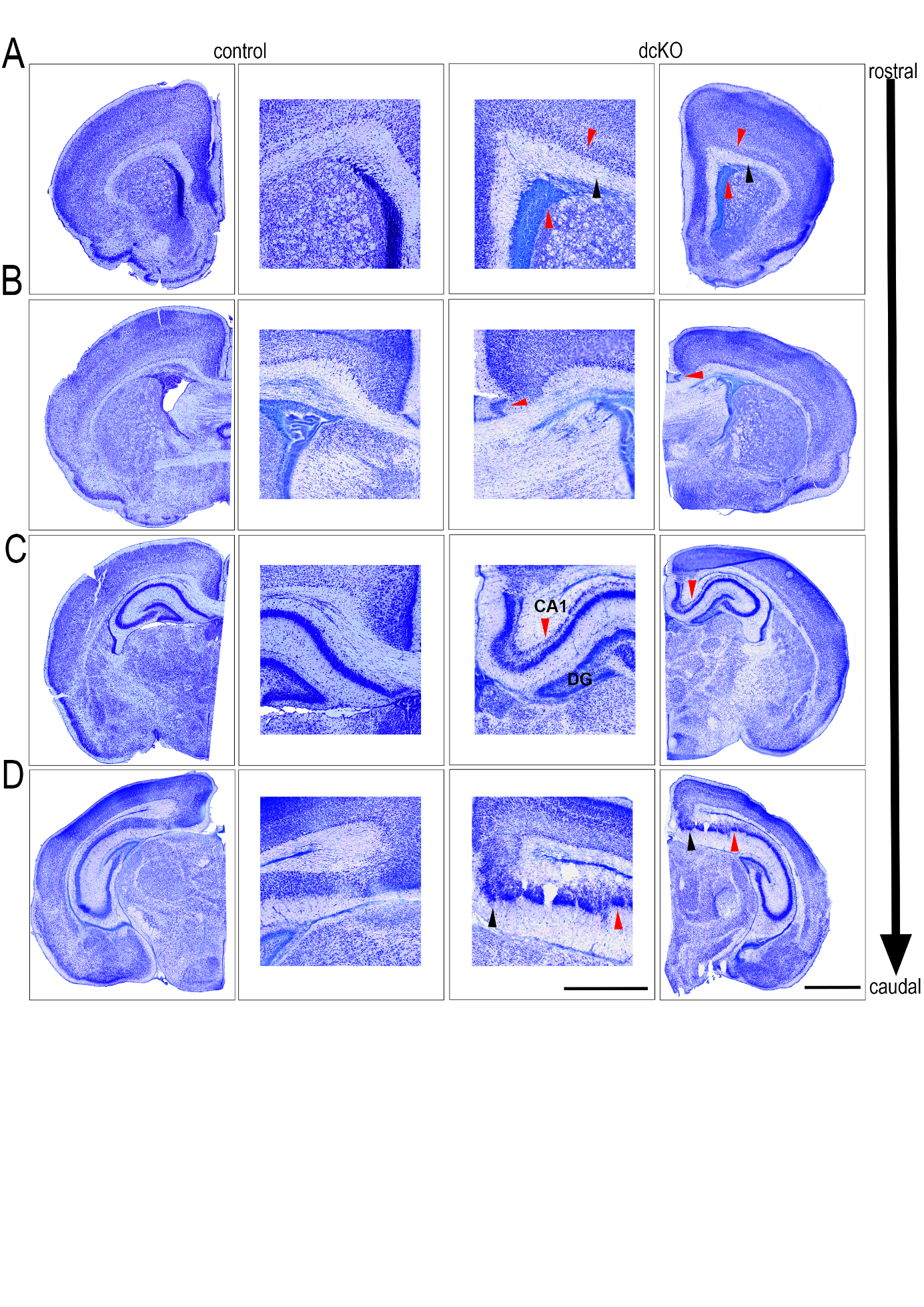


**Figure S4: Nissl stain reveals structural and anatomical malformations in dcKO brains.** (A-D) Cresyl violet (Nissl) stained coronal brain sections from control and dcKO at rostral, mid, and caudal levels at P7. (A) dcKO showed enlarged lateral ventricles (upward red triangle), denser ventricular zone (VZ; downward red triangle), and thickened corpus callosum forceps anterior compared to controls (upward black triangle). (B) The red triangle indicates malformation of the indusium griseum, which appears bulgy and hook-shaped in dcKO mice. Normally, the indusium griseum is a thin layer of neurons oriented parallel to the dorsal surface of the corpus callosum, as observed in control littermates. (C) In dcKO brains, the hippocampus displays a pronounced curved shape, and the CA1 region contains an ectopic layer of cells positioned above the normal CA1 area (downward red triangle). The dentate gyrus (DG) was elongated, with lower cellular density compared to controls. (C) In control animals, a clear boundary is visible between the retrosplenial cortex and the subiculum, whereas in dcKO mice this boundary is absent (higher magnification black upward triangle). In the dcKO subiculum, cells are ectopically densely packed and appear in clusters, while in control animals the cells are homogeneously distributed (higher magnification upward red triangle). Scale bar: 500 µm.


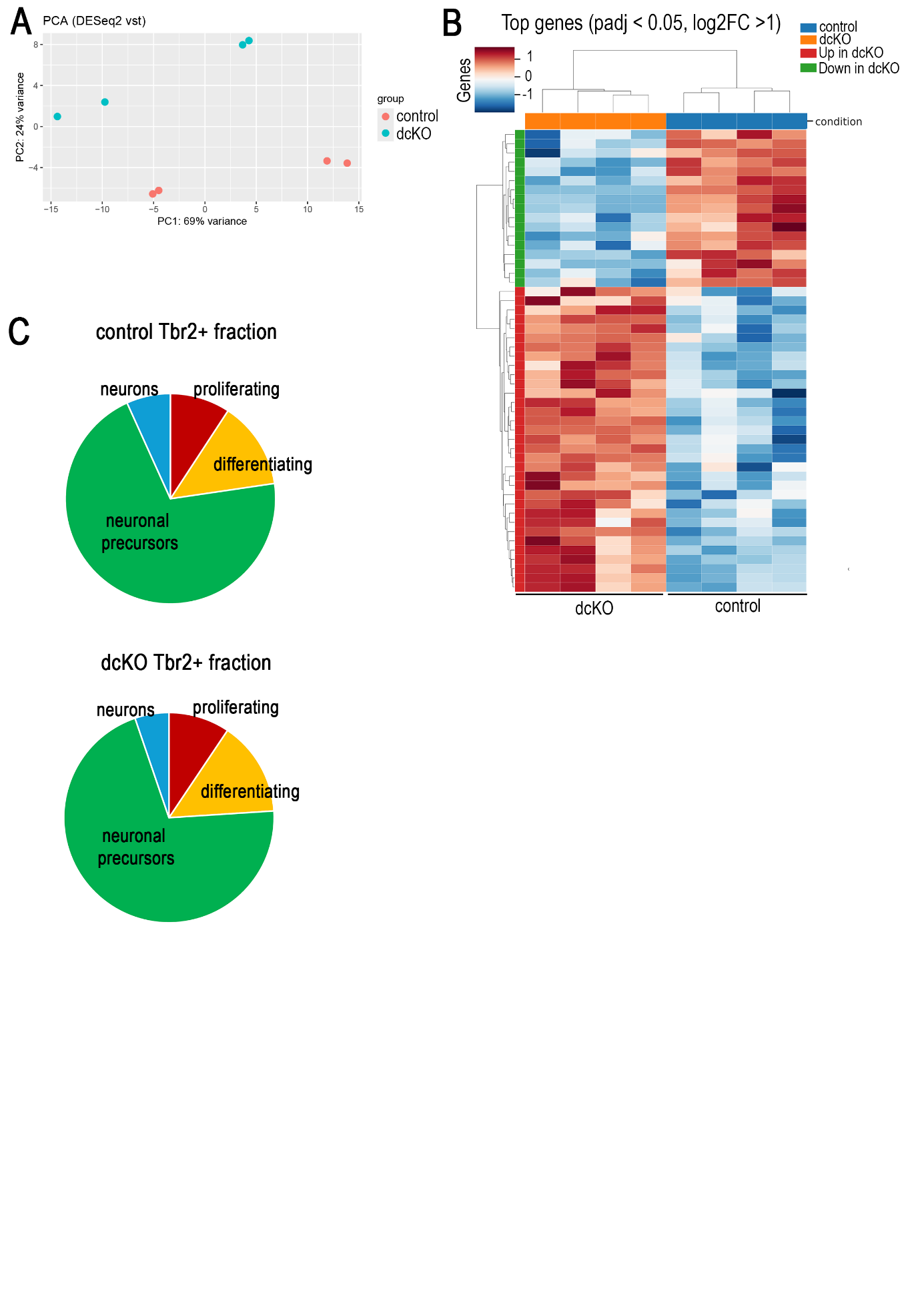


**Figure S5: Bulk RNA- seq. analysis of control vs. dcKO Tbr2+ IPCs: quality control and top gene heatmap**. (A) Principal component analysis (PCA) plot of normalized bulk RNA-seq. data from four technical replicates each of control and dcKO cortices, processed using DESeq2’s variance-stabilizing transformation (VST). PC, principal components. (B) Heatmap of normalized bulk RNA-seq. data from the same samples, generated using deepTools (plotHeatmap). (C) Deconvolution of bulk-RNA-seq data with CIBERSORTx algorithm, determines no changes in the relative contribution of Tbr2+ subpopulations in dcKO relative to control. The top differentially expressed genes are shown (padj < 0.05, log2FC >1).


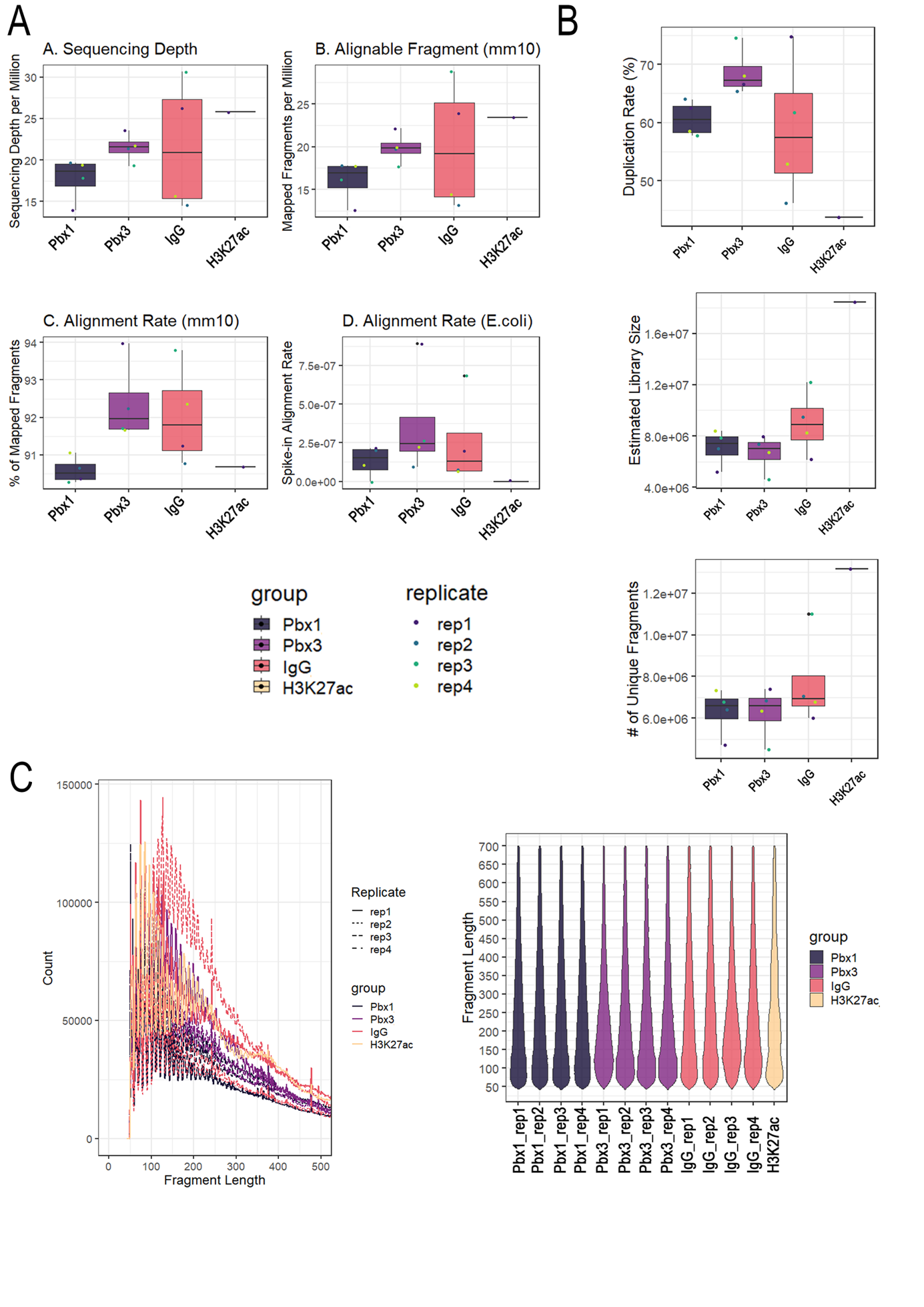


**Figure S6: Quality control of CUT&Tag seq. analysis in control samples at E15.5.**
(A) Align summary for CUT&Tag data from four technical replicates of Pbx1, Pbx3, IgG (negative control) and H3K27ac (positive control). (B) Duplication rate summary from the same four technical replicates. (C) Fragment size distribution from the same four technical replicates.


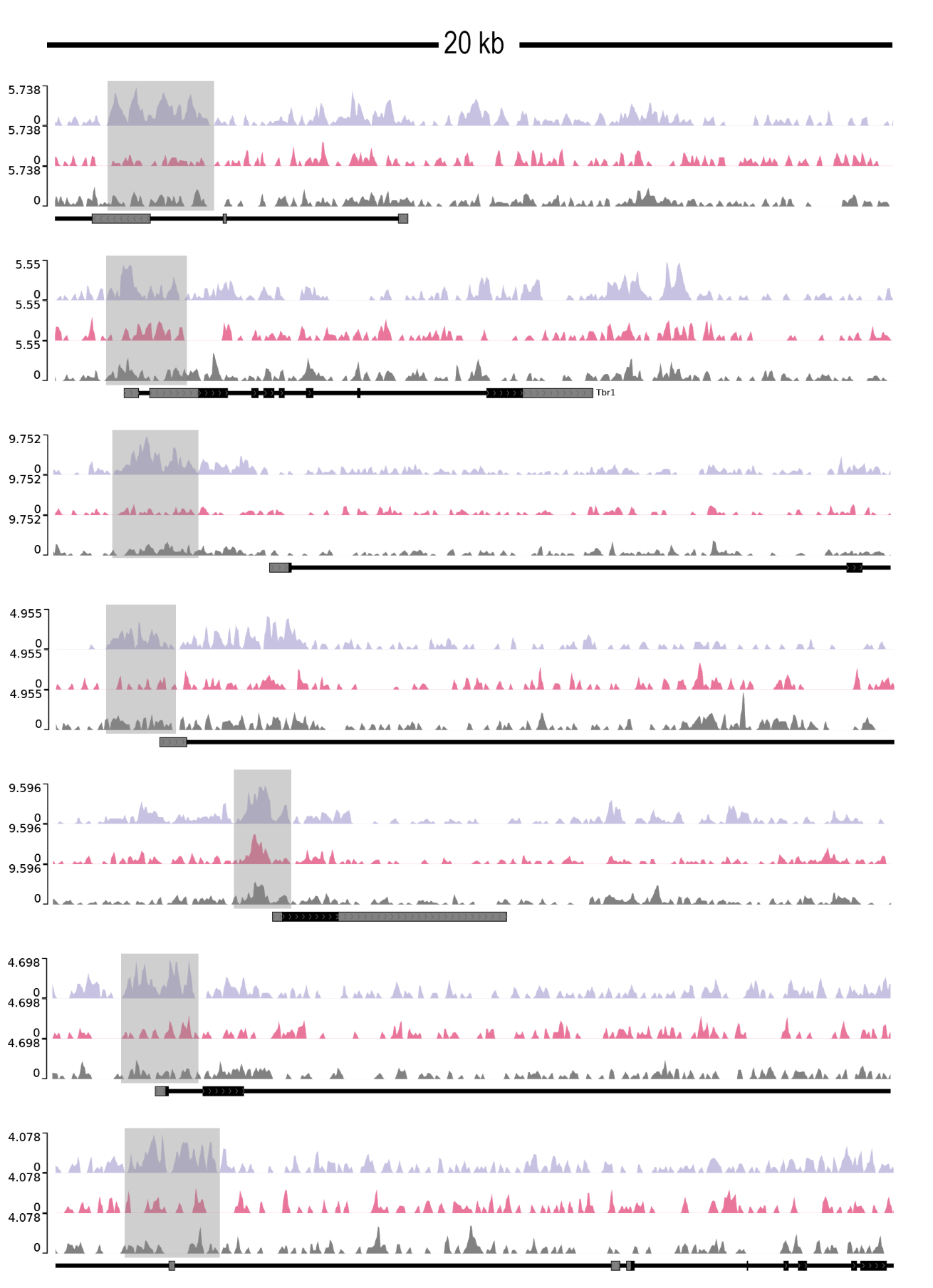


**Figure S7:** **CUT&Tag‑seq genome tracks showing Pbx1/Pbx3 regulated gene loci.** Pbx1 and Pbx3 occupancy were detected at promoter/TSS regions of Satb2, Tbr1, Ctip2, and Rorb (genes upregulated in dcKO P7 cortices), as well as Brn2, Cux1, and L1cam (genes downregulated in dcKO P7 cortices).
